# A Light-Driven In Vitro Enzymatic Biosystem for the Synthesis of α-Farnesene from Methanol

**DOI:** 10.34133/bdr.0039

**Published:** 2024-07-30

**Authors:** Xinyue Gui, Fei Li, Xinyu Cui, Ranran Wu, Dingyu Liu, Chunling Ma, Lijuan Ma, Huifeng Jiang, Chun You, Zhiguang Zhu

**Affiliations:** ^1^ Key Laboratory of Industrial Fermentation Microbiology, Ministry of Education, Tianjin Key Laboratory of Industrial Microbiology, The College of Biotechnology, Tianjin University of Science and Technology, Tianjin 300457, China.; ^2^ Key Laboratory of Engineering Biology for Low-Carbon Manufacturing, TianjinInstitute of Industrial Biotechnology, Chinese Academy of Sciences, Tianjin 300308, China.; ^3^ University of Chinese Academy of Sciences, Beijing 100049, China.

## Abstract

Terpenoids of substantial industrial interest are mainly obtained through direct extraction from plant sources. Recently, microbial cell factories or in vitro enzymatic biosystems have emerged as promising alternatives for terpenoid production. Here, we report a route for the synthesis of α-farnesene based on an in vitro enzyme cascade reaction using methanol as an inexpensive and renewable C1 substrate. Thirteen biocatalytic reactions divided into 2 modules were optimized and coupled to achieve methanol-to-α-farnesene conversion via integration with natural thylakoid membranes as a green energy engine. This in vitro enzymatic biosystem driven by light enabled the production of 1.43 and 2.40 mg liter^−1^ α-farnesene using methanol and the intermediate glycolaldehyde as substrates, respectively. This work could provide a promising strategy for developing light-powered in vitro biosynthetic platforms to produce more natural compounds synthesized from C1 substrates.

## Introduction

Terpenoids, including the sesquiterpene α-farnesene, are an important group of natural compounds with diverse applications in materials, flavors, agriculture, and medicine [1–3]. The natural biosynthetic pathways of terpenoids include the mevalonate (MVA) pathway and the 2-*C*-methyl-d-erythritol 4-phosphate/1-deoxy-d-xylulose 5-phosphate (MEP) pathway. Terpenoids are subsequently synthesized from different numbers of molecules of isopentenyl pyrophosphate (IPP) and dimethylallyl pyrophosphate (DMAPP) generated from these 2 pathways. Usually, plant cells have both the MVA and MEP pathways, while most other organisms, such as gram-negative bacteria, photosynthetic cyanobacteria, and green algae, have only the MEP pathway.

Traditionally, terpenoids are obtained through direct extraction from plant sources, which suffer from low yields and poor purities and fail to meet the needs of human society [4]. The chemical synthesis of structurally complex terpenoids from petrochemicals remains challenging and environmentally unfriendly. Alternatively, microbial cell factories, particularly with the aid of synthetic biology, have emerged as a promising route for the synthesis of industrially important terpenes. Engineered strains such as cyanobacteria, *Escherichia coli*, and *Saccharomyces cerevisiae* have been widely used to produce terpenoids from renewable substrates. For example, the model cyanobacterium *Synechococcus elongatus* PCC7942, with the introduction of an optimized MEP pathway and a heterologous farnesene synthase (FS), produced 4.6 mg liter^−1^ α-farnesene in 7 days from CO_2_ [5]. The introduction of an MVA-based lycopene metabolic pathway to *E. coli* resulted in a lycopene yield of 219.7 mg g^−1^ dry cell weight [6]. Similarly, a rewired *S. cerevisiae* strain produced 190.5 mg liter^−1^ α-farnesene through the combination of the MVA pathway and a soybean-derived FS [7]. Furthermore, with advances in cell-free synthetic biology, in vitro synthetic biosystems have also been shown to successfully produce some terpenoids due to the advantages of an easy pathway design and high modularity [8]. For instance, an enzymatic cascade starting from acetic acid based on the MVA pathway yielded 1.6 g liter^−1^ farnesyl diphosphate (FPP) after optimization [9]. A one-pot cell-free biosystem, including a soluble P450 and a 5-enzyme orthogonal cofactor regeneration module, led to the synthesis of 1 g liter^−1^ nepetalactone [10]. With as many as 27 enzymes, a complicated in vitro biosystem for the conversion of glucose into monoterpenes was constructed, with the production of 12.5 g liter^−1^ limonene and 14.9 g liter^−1^ pinene [11]. These endeavors have indicated that in vitro biosystems may outperform in vivo biosystems in terms of not only pathway design but also product yield.

However, in vitro synthetic biosystems still face challenges pertaining to the likely use of costly and unstable cofactors, as they are unable to self-replenish cofactors as cellular systems do [12]. Currently, 2 methods have been developed to address cofactor regeneration: eliminating the use of cofactors by designing a redox-balanced enzymatic pathway or adding a sacrificial substrate and enzyme to construct a cofactor regeneration module within the constructed pathway [13–15]. Nevertheless, byproduct generation and an additional thermokinetic burden may arise from these approaches. Alternatively, naturally existing thylakoid membranes (TMs), which function as light-powered green engines for the coregeneration of ATP (adenosine triphosphate) and NADPH [reduced form of nicotinamide adenine dinucleotide phosphate (NADP^+^)], have been used. For example, with the aid of TMs, an artificial carbon-fixing cycle, crotonyl-coenzyme A (CoA)/ethylmalonyl-CoA/hydroxybutyryl-CoA (CETCH), was constructed with the production of glycolate at concentrations of up to 47 μM in 90 min by fixing carbon dioxide [16]. For the first time, TMs were applied to produce poly(3-hydroxybutyrate) from acetate in an in vitro synthetic enzymatic biosystem with a light energy conversion of 3.04% [17]. This finding suggests the promise of using TMs to drive more in vitro biosystems.

In recent years, C1 compounds such as carbon monoxide, carbon dioxide, methanol, and methane have become the preferred feedstocks in biomanufacturing due to their natural abundance, low cost, and good sustainability [18]. Considerable efforts have been devoted to the bioconversion of C1 compounds through in vitro biosystems [19]. Valuable chemicals such as n-butanol, ethanol, rare functional sugars, and even starch have been produced from C1 feedstock via naturally or artificially designed enzyme cascades [20–22]. However, the use of C1 feedstock for the in vitro biosynthesis of terpenoids has rarely been reported. Notably, in many terpenoid biosynthesis pathways, acetyl-CoA (AcCoA) is an essential intermediate. Recent work on the conversion of methanol or formaldehyde to AcCoA using an unnatural pathway has been reported [23]. Therefore, this beautiful C1-to-AcCoA pathway could function as a bridge between C1 compounds and the MVA pathway to realize terpenoid biosynthesis with C1 substrates.

Here, we developed an in vitro enzymatic biosystem to synthesize α-farnesene using methanol as the substrate. The system comprising 13 steps catalyzed by 14 enzymes can be separated into 2 synthetic modules to achieve this goal. The 4 regeneration reactions driven by light-powered TMs include 3 ATP regeneration reactions and one NADPH regeneration reaction. This in vitro biosystem is expected to achieve the coupling of light energy input and α-farnesene synthesis from C1 substrates such as methanol. Through enzyme mining and optimization of the biosystem, such as the concentrations of enzymes, TMs, and cofactors, the product yield can be enhanced. This study could provide a promising route for the biosynthesis of α-farnesene. Additionally, the information gained from in vitro biosystems could guide the engineering of photosynthetic cell factories for the synthesis of many other terpenes.

## Materials and Methods

### Chemicals and strains

All chemicals and biochemicals were purchased from Sigma-Aldrich (St. Louis, MO, USA), Thermo Fisher Scientific (Shanghai, China), Sinopharm (Shanghai, China), and Aladdin (Shanghai, China), unless noted otherwise. Primers and synthesized genes were obtained from GENEWIZ (Suzhou, China). Aminex HPX-87H columns were purchased from Bio-Rad (Hercules, USA). High-affinity Ni-NTA resins were purchased from GE Healthcare Life Sciences (USA). Alcohol oxidase (AOX) was purchased from Sigma-Aldrich. Catalase (Cat) was purchased from Auwitkey (Suzhou, China).

### Construction of plasmids

The genes encoding the target proteins, including *GALS*, *EcGCL*, *ACPS*, *PTA*, *PhaA*, *Hmgs*, *Hmgr*, *Mvk*, *Pmvk*, *Mdc*, *IDI*, *ISPA MdFs*, and *AFS*, were synthesized and constructed in the pET28a plasmid, as shown in Table S3. The plasmid primers for all enzymes used in the study are listed in Table S4. The DNA sequences of the relevant enzymes are shown in Table S5. All the plasmids were transformed into the *E. coli* strain DE3 (BL21) for overexpression.

### Protein synthesis and purification

Strains of *E. coli* BL21(DE3) harboring the relevant expression plasmid were incubated in 5 ml of LB medium supplemented with 50 μg ml^−1^ kanamycin at 37°C until the OD_600_ reached approximately 0.6 to 0.8, which was induced by the addition of IPTG (isopropyl-β-d-thiogalactopyranoside) at a final concentration of 0.1 mM, followed by an incubation at 16°C for 18 to 20 h. After the collection of the cells by centrifugation at 5,000 rpm, the precipitate was resuspended in buffer A (50 mM Hepes, 50 mM NaCl, pH 7.5). *Ec*GCL was suspended in 100 mM tris-HCl buffer containing 300 mM KCl and 5 mM magnesium sulfate (pH 7.5). The cell suspensions were lysed in a high-pressure homogenizer, and the supernatant was collected by centrifugation at 8,000 rpm for 30 min. The supernatant was transferred to a Ni-NTA resin column, washed with 3 to 5 CVs (column volumes) of buffer B (30 mM imidazole in buffer A), and eluted with buffer C (500 mM imidazole in buffer A). Specifically, PTA and IDI were eluted with buffer C containing 400 mM NaCl to maintain protein activity. GALS_F397YC398M_ was eluted with 500 mM imidazole in 50 mM Hepes buffer containing 5 mM magnesium sulfate (pH 7.5). Purified target proteins were concentrated in ultrafiltration tubes and subjected to 12% SDS–polyacrylamide gel electrophoresis (PAGE). Protein concentrations were determined using the Bradford method with bovine serum albumin as the standard.

### Enzymatic activity assays

The enzyme activities of GALS_F397YC398M_ and *Ec*GCL were determined in the same manner. The reaction mixture comprised 50 mM potassium phosphate buffer (pH 7.4), 5 mM MgSO_4_, 0.5 mM ThDP, 50 μg ml^−1^ glycerol dehydrogenase, 1 mM NADH [reduced form of nicotinamide adenine dinucleotide (oxidized form)], and different concentrations of formaldehyde. The reaction was started by adding GALS_F397YC398M_ or *Ec*GCL at 37°C, and an initial linear decrease in absorbance was observed at 340 nm [24]. The kinetic parameters *k*_cat_ and *K*_m_ of *Ec*GCL were estimated according to the Michaelis–Menten equation using GraphPad Prism 5 software.

#### Enzyme activity assay for ACPS

The reaction mixture (200 μl) contained 50 mM potassium phosphate buffer (pH 7.5), 5 mM MgSO_4_, 1 mM ThDP, 10 mM glycolaldehyde, 1 mM adenosine diphosphate (ADP), 0.2 mg ml^−1^ acetate kinase, 5 U hexokinase, 2.5 U glucose-6-phosphate dehydrogenase, 1 mM NADP^+^, and 10 mM glucose. ACPS was added to the reaction system at a concentration of 0.5 mg ml^−1^, and the reaction was performed at 37°C. NADPH production was detected at 340 nm [23].

#### Enzyme activity assay for PTA

For the determination of PTA activity, the inverse reaction method was used. The reaction mixture consisted of 100 mM tris-HCl (pH 7.2), 5 mM MgCl_2_, 5 mM KH_2_PO_4_, 0.1 mM 5,5′-dithiobis-(2-nitrobenzoic acid), and 0.1 mM AcCoA. The reaction was performed at 55°C, and the absorbance was measured at 412 nm (ɛ_412_ = 13.5 mM^−1^ cm^−1^) [25].

### Isolation of TMs

TMs were isolated from *Spinacia oleracea* by Percoll/sucrose gradient centrifugation [26,27]. Fresh spinach leaves were harvested and blended with buffer A [50 mM Hepes-KOH, pH 7.6, 330 mM sorbitol, 5 mM MgCl_2_, and 0.1% (w/v) bovine serum albumin], and then the homogenate was filtered through 8 layers of gauze. The filtrate was centrifuged at 4,000 rpm for 10 min. The precipitates were collected and suspended in buffer B (50 mM Hepes-KOH, pH 7.6, 300 mM sorbitol, 5 mM MgCl_2_, and 10 mM sodium L-ascorbate) and then overlaid on a 80/40% Percoll gradient (80%:80% v/v Percoll, 300 mM sucrose, 10 mM sodium L-ascorbate, and 60 mM Mops-KOH, pH 7.6 and 40%:40% v/v Percoll, 300 mM sucrose, 10 mM sodium L-ascorbate, and 25 mM Mops-KOH, pH 7.6). The bands of the TMs were harvested by centrifugation at 4,000 rpm for 10 min and washed twice with buffer B. The sediment was subsequently suspended in buffer C (10 mM Hepes-KOH, pH 7.6, 10 mM sodium L-ascorbate, 10 mM MgCl_2_, and 10% dimethyl sulfoxide) and stored at −80°C. Before use, the TMs were washed 2 times with buffer D (10 mM Hepes-KOH, pH 7.6, 300 mM sorbitol, 10 mM MgCl_2_, and 10 mM sodium L-ascorbate).

### ATP and NADPH production rates

Thylakoid activity was assayed by monitoring NADPH production at 340 nm in a 0.6-ml reaction volume. The reaction consisted of 50 mM Hepes-KOH (pH 7.8), 3 mM ADP, various concentrations of ferredoxin (Fdx), 5 mM K_2_HPO_4_, 3 mM NADP^+^, 10 mM sodium L-ascorbate, 10 mM KCl, 5 mM MgCl_2_, and TMs of 5 μg of chlorophyll equivalents. The samples were illuminated with white light at different intensities. ATP production was also measured using an ATP Assay Kit (Grace, China). Note that all values shown in this work are the means of triplicate measurements. The error bars represent standard deviations.

### Glycolaldehyde synthesis from methanol in vitro

Using 30 mM methanol as the initial substrate, 0.7 mg ml^−1^ AOX, 0.4 mg ml^−1^ Cat, and 2 mg ml^−1^ GALS_F397YC398M_ were added to 100 mM Hepes buffer (pH 7.5) containing 5 mM MgSO_4_ and 1 mM ThDP in a 500-μl reaction system and incubated for 6 h at 37°C. One hundred microliters of samples was collected at 2-h intervals, and the reaction was terminated with 10% sulfuric acid. After centrifugation at 13,000 rpm for 10 min, the samples were analyzed using high-performance liquid chromatography (HPLC). The HPLC instrument was equipped with an Aminex HPX-87H column, and the HPLC conditions were set at 40°C with a mobile phase of 5 mM sulfuric acid and a flow rate of 0.6 ml min^−1^ [24].

### In vitro synthesis of α-farnesene from glycolaldehyde

The reaction system for the synthesis of α-farnesene from glycolaldehyde in vitro without the addition of TMs contained 50 mM sodium phosphate buffer (pH 7.4), 3 mM glycolaldehyde, 0.5 mg ml^−1^ each protein, 1 mM NADPH, 5 mM ATP, 30 mM KCl, 1 mM tris(2-carboxyethyl)phosphine (TCEP), and 10 mM MgCl_2_. The reaction was incubated at room temperature for 120 min.

The reaction system for the synthesis of α-farnesene from glycolaldehyde in vitro was driven by light-powered TMs in a solution containing 50 mM sodium phosphate (pH 7.4), 30 mM glycolaldehyde, 1 mM CoA, 5 mM ADP, 1 mM NADP^+^, 5 μM Fdx, 3.5 mg ml^−1^ ACPS, 0.5 mg ml^−1^ PTA, and other ration-defined enzymes (the molar ratio of PhaA/Hmgs/Hmgr/Mvk/Pmvk/Mdc/IDI/ISPA/AFS was 1:10:2:5:5:2:5:2:2), 35 mM KCl, 1 mM TCEP, 15 mM MgCl_2_, 0.5 M betaine, 10 mM sodium L-ascorbate, 10 mM K_2_HPO_4_, and 30 μg ml^−1^ TMs. The reaction was incubated under 50 μmol photons m^−2^ s^−1^ of illumination for 6 or 12 h.

### Analytical method for α-farnesene

α-Farnesene was extracted with dodecane. The samples were mixed 1:1 with dodecane, vortexed, and centrifuged at 12,000 rpm for 5 min. The dodecane phase was quantified using a gas chromatograph–mass spectrometer (GC–MS, Thermo Fisher Scientific) equipped with a TraceGOLD TG-5SILMS column (30 m × 0.25 mm × 0.25 μm) and an Orbitrap Exploris GC 240 mass spectrometer (Thermo Fisher Scientific). The GC program was as follows: initial temperature of 60°C, hold for 1 min; linear ramp to 300°C in 12 min, hold for 10 min. The data were acquired in full-scan mode [30 to 350 mass/charge ratio (*m/z*)].

### Optimization of module 1

Hepes buffer (pH 7.5), sodium phosphate buffer (pH 7.4), and potassium phosphate buffer (pH 7.4) used for the reaction of module 1 at 37°C were optimized first. The initial reaction mixture (0.5 ml) contained 50 or 100 mM buffer, 30 mM methanol, 0.7 mg ml^−1^ AOX, 0.4 mg ml^−1^ Cat, 2 mg ml^−1^ GALS_F397YC398M_/*Ec*GCL, 5 mM MgSO_4_, and 1 mM ThDP. Subsequently, the concentration of methanol varied from 10 to 30 mM and was optimized. The concentrations of the enzymes used to load AOX and *Ec*GCL were varied from 0.5 to 3 U ml^−1^ for optimization.

### Optimization of module 2

The reaction conditions for the synthesis of α-farnesene from glycolaldehyde without the addition of TMs were optimized. The ACPS loading was varied from 0.5 to 5 mg ml^−1^, while PTA was loaded at 0.5 mg ml^−1^, and the other enzymes, including PhaA, Hmgs, Hmgr, Mvk, Pmvk, Mdc, IDI, ISPA, and AFS, were loaded at a molar ratio of 1:10:2:5:5:2:5:2:2 in 50 mM sodium phosphate buffer (pH 7.4) containing 20 mM glycolaldehyde, 1 mM NADPH, 5 mM ATP, 30 mM KCl, 1 mM TCEP, and 10 mM MgCl_2_ at room temperature. Under the optimal conditions for ACPS, the PTA concentration was varied from 0.5 to 3.5 mg ml^−1^. The concentration of glycolaldehyde was optimized to vary from 10 to 30 mM. PhaA, Hmgs, Hmgr, Mvk, Pmvk, Mdc, IDI, ISPA, and AFS were optimized in 5-fold increments using 1 mM AcCoA as the substrate.

The reaction conditions for the synthesis of α-farnesene from glycolaldehyde with TMs were optimized based on an initial reaction system containing 50 mM sodium phosphate buffer (pH 7.4), 20 mM glycolaldehyde, 1 mM NADPH, 5 mM ATP, 30 mM KCl, 1 mM TCEP, 10 mM MgCl_2_, 3.5 mg ml^−1^ ACPS, 0.5 mg ml^−1^ PTA, 30 μg ml^−1^ TMs, 5 μM Fdx, 5 mM ADP, and 1 mM NADP^+^. The enzymes PhaA, Hmgs, Hmgr, Mvk, Pmvk, Mdc, IDI, ISPA, and AFS were loaded at a molar ratio of 1:10:2:5:5:2:5:2:2. The concentration of ADP for optimization varied from 0.5 to 3 mM. Under the optimal conditions for ADP, the concentration of NADP^+^ was subsequently optimized from 0.5 to 3 mM. After the optimal concentrations of ADP and NADP^+^ were both determined, the concentration of TMs was varied from 30 to 60 μg ml^−1^ for optimization, while the concentration of Fdx was adjusted from 5 to 20 μM. The reaction was incubated under 50 μmol photons m^−2^ s^−1^ of illumination at room temperature. The light intensity was changed from 50 to 300 μmol photons m^−2^ s^−1^ while maintaining the other conditions constant.

### Analytical method for methanol

The residual concentration of methanol after the reaction was detected using a SIEMAN biosensor analyzer (Shenzhen, China). The reaction was terminated with 10% sulfuric acid and centrifuged at 13,000 rpm for 10 min to remove the protein, and the reaction samples were diluted to a concentration of no more than 0.2 g liter^−1^ on the biosensor.

## Results and Discussion

### In vitro light-driven biosystem design for α-farnesene synthesis

**Fig. 1.**
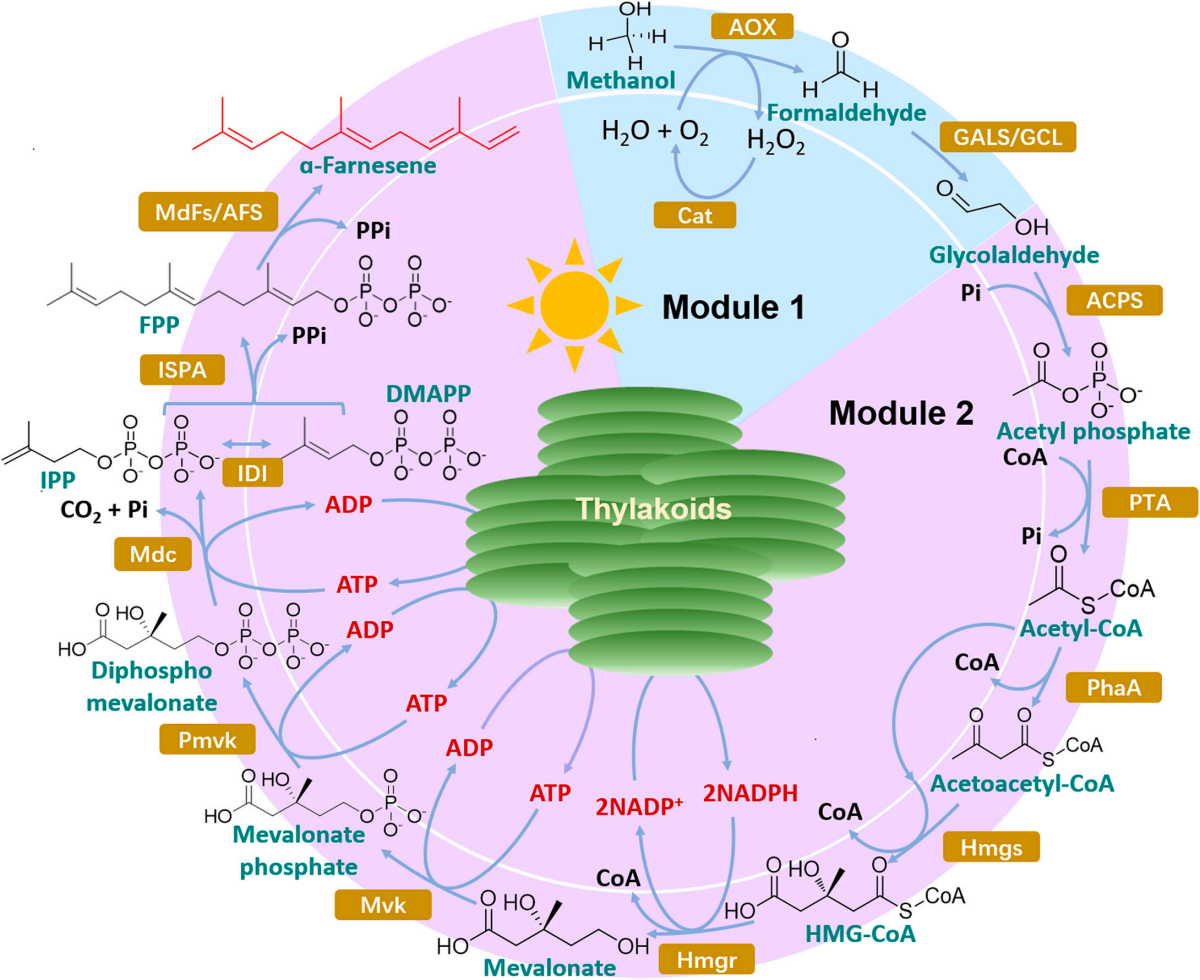
The designed light-powered in vitro biosystem for α-farnesene synthesis. The pathway from methanol to α-farnesene includes the recycling of ATP and NADPH using natural thylakoid membranes. Pi, orthophosphate; CoA, coenzyme A; NADP(H), nicotinamide adenine dinucleotide phosphate; ATP, adenosine triphosphate; ADP, adenosine diphosphate; PPi, diphosphate; AOX, alcohol oxidase; Cat, catalase; GALS/GCL, glycolaldehyde synthase; ACPS, acetyl-phosphate synthase; PTA, phosphate acetyltransferase; PhaA, acetoacetyl-CoA synthase; Hmgs, 3-hydroxy-3-methylglutaryl-CoA synthase; Hmgr, 3-hydroxy-3-methylglutaryl-coenzyme A reductase; HMG-CoA, 3-hydroxy-3methylglutaryl-CoA; Mvk, mevalonate kinase; Pmvk, phosphomevalonate kinase; Mdc, diphosphomevalonate decarboxylase; IDI, isopentenyl diphosphate delta-isomerase; IPP, isopentenyl pyrophosphate; DMAPP, dimethylallyl diphosphate; ISPA, farnesyl diphosphate synthase; FPP, farnesyl pyrophosphate; MdFs/AFS, α-farnesene synthase.

Two metabolic pathways (the MVA and MEP pathways) are known for the biosynthesis of α-farnesene. In our design, the MVA pathway was chosen because it can avoid the cofactor regeneration problem in the MEP pathway and has a key intermediate, AcCoA, which can be synthesized from methanol using the SACA pathway [23]. The multienzyme pathway involves a 13-step biocatalytic reaction and 14 enzymes from different species (Fig. 1). This pathway is currently the shortest route for the synthesis of α-farnesene from methanol in vitro and has a high carbon economy; 18 molecules of methanol are required for the biosynthesis of one molecule of α-farnesene, while 3 carbons are released in the form of CO_2_.The information on the enzymes used in this work is shown in Table S1. Specifically, AOX catalyzes the oxidation of methanol to formaldehyde while producing hydrogen peroxide as a byproduct, which is further broken down into water and oxygen by Cat. Then, under the action of glycolaldehyde synthase (GALS_F397YC398M_ or *Ec*GCL), 2 molecules of formaldehyde are condensed into one molecule of glycolaldehyde, and acetyl phosphate synthase (ACPS) catalyzes glycolaldehyde to synthesize acetyl phosphate, followed by phosphate acetyltransferase (PTA) catalysis of acetyl phosphate to synthesize AcCoA, which can be coupled with the MVA pathway. The common precursor of terpenoids, IPP, can be synthesized through the MVA pathway, and under the action of isopentenyl diphosphate delta isomerase (IDI), dimethylallyl diphosphate (DMAPP) is formed. Next, DMAPP combines with IPP to form farnesyl pyrophosphate (FPP), the direct precursor of sesquiterpenoids, which is further catalyzed by α-farnesene synthase to synthesize α-farnesene. All reactions and the standard Gibbs free energy change (Δ*G′^0^*) of each enzymatic reaction are listed in Table S2. The overall Δ*G′^0^* was calculated to be −426.8 kJ mol^−1^ (Fig. S1). This thermodynamic analysis indicated that a theoretical stoichiometry of 5.6% for methanol to α-farnesene could be achieved using this in vitro biosystem. The TM was chosen as a green engine using a clean electron source (e.g., H_2_O) with the capability of spatiotemporally coregeneration of ATP and NADPH driven by light to address the energy supply issue, such as the recycling of ATP and NADPH. This light-driven method can overcome the shortcomings of the traditional cofactor regeneration method, which normally requires sacrificial substrates (e.g., glucose or formate), accumulates byproducts, and reduces the atomic economy [28–30].

GALS_F397YC398M_, ACPS, PTA, PhaA, Hmgs, Hmgr, Mvk, Pmvk, Mdc, IDI, ISPA, and MdFs were overexpressed and purified to prove the feasibility of our design, as shown in Fig. S2. For the conceptual assay, 30 mM methanol was used as the substrate, and NADPH and ATP were added directly for α-farnesene synthesis. However, α-farnesene was not produced from methanol in the one-pot enzymatic reaction (data not shown). Enzymes in the MVA pathway may be affected by methanol molecules or the accumulation of intermediate formaldehyde molecules. We divided the pathway into 2 independent modules to address these possible issues: module 1 (glycolaldehyde synthesis from methanol) and module 2 (α-farnesene synthesis from glycolaldehyde). Moreover, methanol can be obtained from carbon dioxide via multiple chemical or biological methods. In this sense, the synthesis of terpenes from C1 compounds such as methanol or even carbon dioxide could be achieved [31].

### Synthesis of glycolaldehyde from methanol

**Fig. 2.**
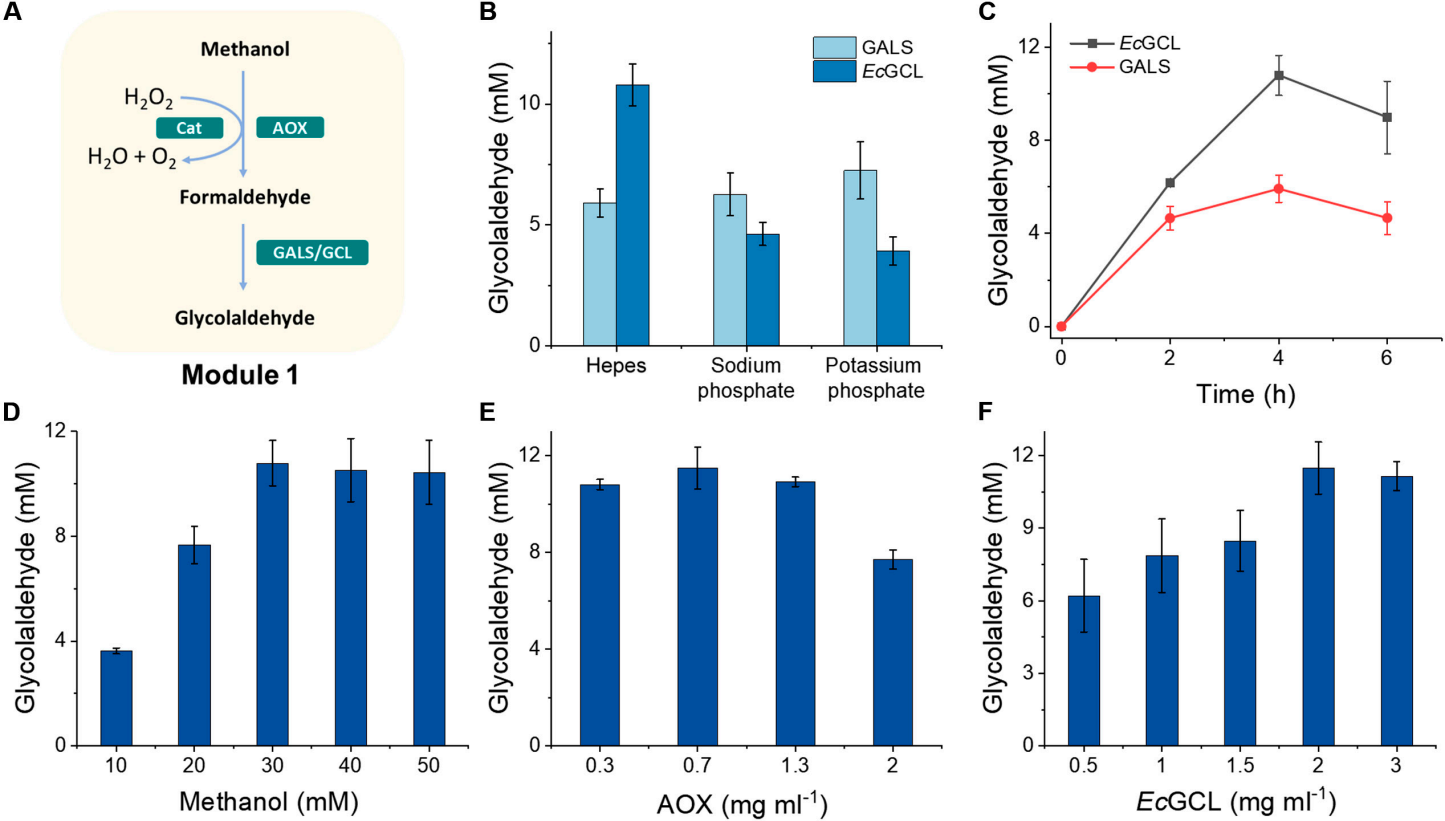
Optimization of module 1. (A) Reaction diagram of module 1. (B) Different buffers used for glycolaldehyde synthesis from methanol catalyzed by GALS_F397YC398M_ or *Ec*GCL. (C) Profiles of glycolaldehyde yield from methanol catalyzed by GALS_F397YC398M_ and *Ec*GCL. The reaction was performed at 37°C in 100 mM Hepes buffer (pH 7.5) with 30 mM methanol, 0.7 mg ml^−1^ AOX, 2 mg ml^−1^ GALS_F397YC398M_, or *Ec*GCL. (D) Optimization of the methanol concentration in 100 mM Hepes buffer (pH 7.5) containing 0.7 mg ml^−1^ AOX and 2 mg ml^−1^ *Ec*GCL at 37°C. (E) Optimization of the AOX concentration from 0.3 to 2 mg ml^−1^ in 100 mM Hepes buffer (pH 7.5) containing 30 mM methanol and 2 mg ml^−1^ *Ec*GCL at 37°C. (F) Optimization of the *Ec*GCL concentration from 0.5 to 3 mg ml^−1^ in 100 mM Hepes buffer (pH 7.5) containing 30 mM methanol and 0.7 mg ml^−1^ AOX at 37°C.

For the dynamic modulation of module 1, AOX, Cat, and GALS_F397YC398M_ were used to convert methanol to glycolaldehyde (Fig. 2A). The standard curve of glycolaldehyde is shown in Fig. S3. Initially, GALS_F397YC398M_, a mutant of benzoylformate decarboxylase (BFD) from *Pseudomonas putida*, was proposed for the condensation of formaldehyde [23]. Zhang et al. [24] increased the *k*_cat_ of GALS_F397YC398M_ by approximately 160-fold and improved the catalytic efficiency by approximately 70-fold compared to that of the original enzyme. In our work, 6 mM glycolaldehyde was obtained from 30 mM methanol in 100 mM Hepes buffer (pH 7.5). Phosphate buffers were also tested to increase the conversion efficiency. This result suggested that potassium phosphate buffer was optimal for GALS_F397YC398M_, yielding 7.3 mM glycolaldehyde from 30 mM methanol (Fig. 2B). This result indicated that the methanol-to-glycolaldehyde conversion efficiency was approximately 50%. Later, a mutant of glyoxylate carboligase (*Ec*GCL) was found to convert formaldehyde to glycolaldehyde [32]. This *Ec*GCL produced 31 mM glycolaldehyde from 75 mM formaldehyde, with a higher substrate affinity than GALS_F397YC398M_ (*K*_m_: 37 mM versus 170 mM). As a result, we expressed *Ec*GCL in *E. coli* and obtained a *K*_m_ value of 29.4 mM, which is consistent with the literature (Fig. S4). Compared with GALS_F397YC398M_, *Ec*GCL had a higher formaldehyde conversion efficiency (Fig. 2C). In a 100 mM Hepes solution (pH 7.5) containing 1.3 mg ml^−1^ AOX, 0.4 mg ml^−1^ Cat, 5 mM MgSO_4_, and 1 mM ThDP, *Ec*GCL could synthesize 10.8 mM glycolaldehyde from 30 mM methanol, which was 1.8-fold greater than that obtained from GALS_F397YC398M_. Then, we further optimized the methanol concentration for this reaction. The yield of glycolaldehyde increased with increasing methanol concentrations from 10 to 30 mM. However, higher methanol concentrations, such as 40 or 50 mM, had no effect on the yield of glycolaldehyde (Fig. 2D). Finally, AOX and *Ec*GCL were optimized, and AOX at 0.7 mg ml^−1^ and *Ec*GCL at 2 mg ml^−1^ were found to be the optimal concentrations (Fig. 2E and F). After the optimization of module 1, 11.4 mM glycolaldehyde could be obtained from 23 mM methanol consumed, representing a molar conversion efficiency of 77% (Fig. S5). To this end, subsequent experiments were performed using 30 mM methanol, 0.7 mg ml^−1^ AOX, 0.4 mg ml^−1^ Cat, and 2 mg ml^−1^ *Ec*GCL in a 100 mM Hepes solution containing 5 mM MgSO_4_ and 1 mM ThDP.

### Synthesis of α-farnesene from glycolaldehyde

**Fig. 3.**
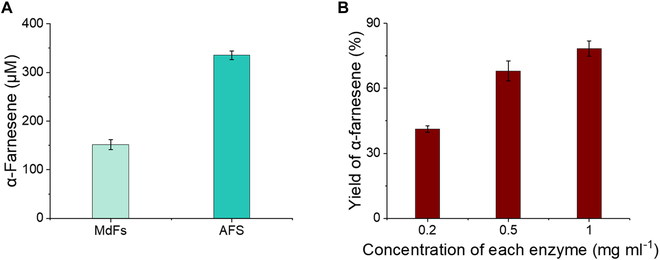
Yield of α-farnesene from the biosystem using MdFS or AFS with IPP as the substrate. (A) MdFS or AFS was used for the synthesis of α-farnesene in 100 mM Hepes buffer (pH 7.5) containing 2 mM IPP, 0.5 mg ml^−1^ MdFS, or AFS at room temperature. (B) Concentrations of 0.2 mg ml^−1^, 0.5 mg ml^−1^, or 1 mg ml^−1^ of all 3 enzymes (IDI, ISPA, and AFS) used for the synthesis of α-farnesene in 100 mM Hepes buffer (pH 7.5) supplemented with 1 mM IPP at room temperature.

In a preliminary experiment, we found that *Malus domestica* α-farnesene synthase (MdFs) from our laboratory stock was poorly expressed in *E. coli* and was difficult to purify. After searching the database and references, we found another gene sequence for *M. domestica subsp. chinensis*-derived α-farnesene synthase (AFS, UniProt No. Q84LB2) [33]. When constructed in pET28a and transferred into BL21 cells for overexpression, AFS was easily overexpressed but the cells contained many inclusion bodies. Unfortunately, after trying to reduce the induction temperature and IPTG concentration with little success, we sought to add the molecular chaperone small ubiquitin-like modifier (SUMO) to the N-terminus to assist in the correct folding of AFS [34–36]. The expression level of AFS in *E. coli* was higher than that of MdFs (Fig. S6). The standard curve of α-farnesene is shown in Fig. S7. When using 2 mM IPP as a substrate and 0.5 mg ml^−1^ IDI, ISPA, and MdFs/AFS, the yield of AFS was greater than that of MdF, with an α-farnesene yield of 336 μM versus 152 μM (Fig. 3A). The yield was further increased to approximately 80% by adjusting the concentrations of all 3 enzymes (IDI, ISPA, and AFS) to 1 mg ml^−1^, with 1 mM IPP used as a substrate (Fig. 3B).

**Fig. 4.**
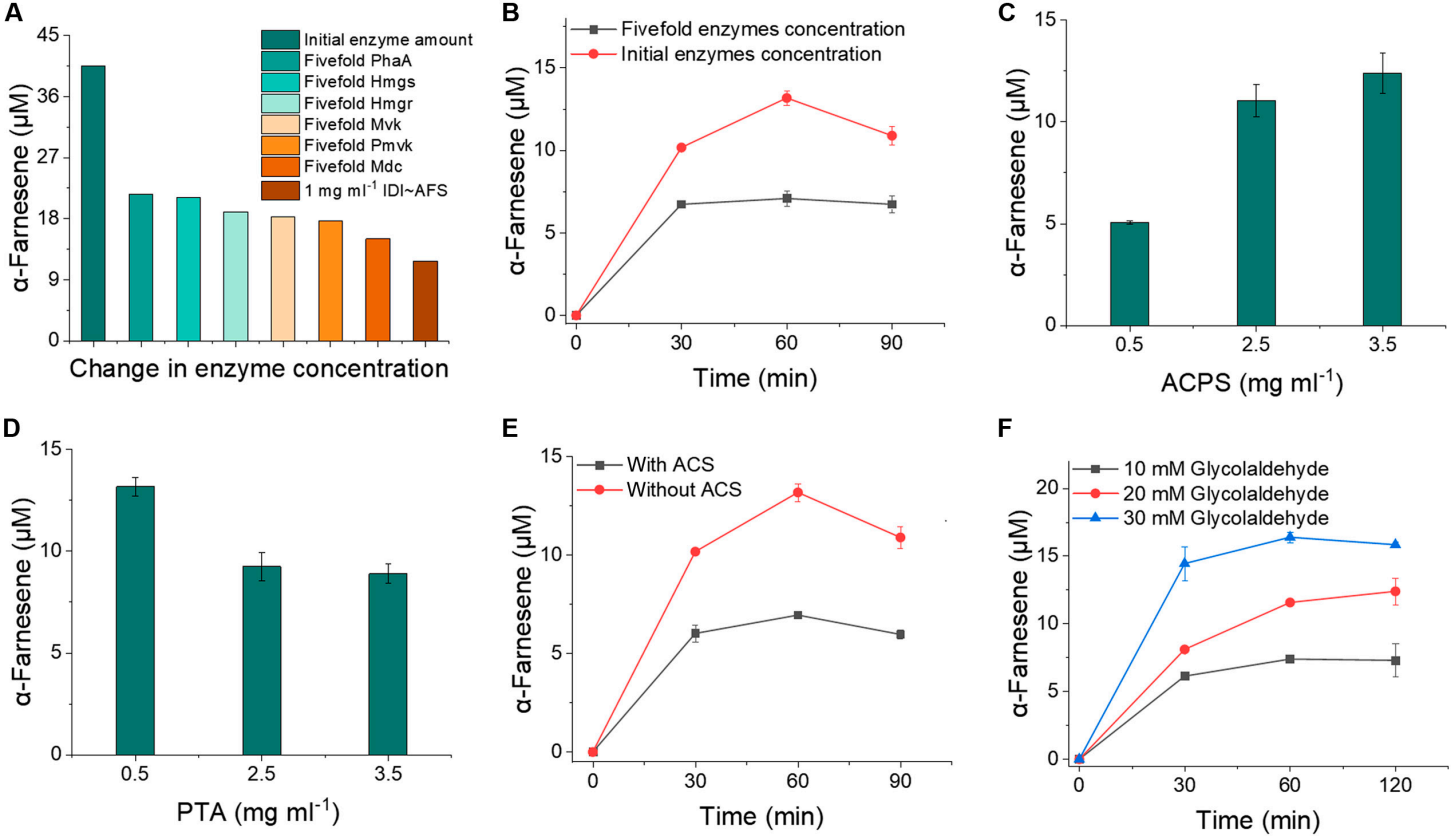
Optimization of module 2. (A) Optimization of PhaA, Hmgs, Hmgr, Mvk, Pmvk, and Mdc concentrations using 1 mM AcCoA as a substrate. These 6 enzymes were added at an original ratio of 1:10:2:5:5:2, and IDI, ISPA, and AFS were added at 1 mg ml^−1^. (B) Fivefold increases in the original ratios of PhaA, Hmgs, Hmgr, Mvk, Pmvk, Mdc, IDI, ISPA, and AFS; 1 mM AcCoA was used as the substrate. (C) Optimization of ACPS. The concentration of ACPS was varied from 0.5 to 5.0 mg ml^−1^ using 20 mM glycolaldehyde as the substrate. (D) Optimization of PTA. The concentration of PTA was varied from 0.5 to 3.5 mg ml^−1^ using 20 mM glycolaldehyde as the substrate. (E) Profile of the α-farnesene yield after the addition of 2 mg ml^−1^ ACS. (F) Profile of the α-farnesene yield in the presence of different concentrations of glycolaldehyde under optimized conditions.

Zhu et al. [37] previously optimized the ratios of MVA pathway enzymes and 3 additional enzymes for the synthesis of α-farnesene from AcCoA. They controlled the molar ratios of PhaA, Hmgs, Hmgr, Mvk, Pmvk, Mdc, IDI, ISPA, and AFS to 1:10:2:5:5:2:5:2:2 in the presence of 1 mM AcCoA. As a result, the amount of α-farnesene increased by more than 6-fold compared with that under other conditions. Therefore, we used this enzyme ratio and 1 mM AcCoA as a substrate and produced 40.5 μM α-farnesene, consistent with the literature (Fig. 4A). Next, we increased the amounts of PhaA, Hmgs, Hmgr, Mvk, Pmvk, and Mdc 5-fold to determine whether the yield of α-farnesene also increased. IDI, ISPA, and AFS were added at a concentration of 1 mg ml^−1^, as suggested in the section above. The results indicated that the highest level of α-farnesene was achieved by adding these 9 enzymes in the specified proportions (Fig. 4A). Changing the concentrations of any of the MVA pathway enzymes resulted in decreased α-farnesene production. Notably, Hmgr was reported to be a rate-limiting enzyme in the MVA pathway [38]. However, we obtained a reduced α-farnesene yield when the concentration of this enzyme was increased. In addition, when the concentrations of PhaA, Hmgs, Hmgr, Mvk, Pmvk, Mdc IDI, ISPA, and AFS were increased simultaneously by a factor of 5, the α-farnesene yield decreased significantly (Fig. 4B). These results indicated that excess amounts of enzymes were not always beneficial, at least in our case.

The loading of ACPS was optimized to vary from 0.5 to 3.5 mg ml^−1^ to evaluate the yield of α-farnesene from glycolaldehyde, and the highest production of α-farnesene was achieved at an ACPS concentration of 3.5 mg ml^−1^ (Fig. 4C). However, the α-farnesene yield decreased when the loading of PTA was varied from 0.5 to 3.5 mg ml^−1^ (Fig. 4D). This result was probably because PTA may interfere with the normal cascade by transforming other intermediates (e.g., acetyl-phosphate to acetic acid). Since some intermediates, such as 3-hydroxy-3-methylglutaryl coenzyme A (HMG-CoA), mevalonate phosphate, and diphosphomevalonate, were not available, no further tests were performed.

As PTA can produce acetic acid as a byproduct during the synthesis of AcCoA from acetyl-phosphate, we hypothesized that the presence of acetic acid may hinder the conversion of glycolaldehyde to α-farnesene. A reaction including 20 mM glycolaldehyde, 5 mM MgCl_2_, 10 mM KCl, 1 mM ThDP, and 1 mM CoA in a 50 mM sodium phosphate solution (pH 7.4) was performed with 0.5 or 2.5 mg ml^−1^ PTA. The standard curve of sodium acetate and the amount of acetic acid in the samples were monitored using HPLC (Fig. S8). The amount of acetic acid produced decreased at higher concentrations of PTA (Fig. S9). We reasoned that as the concentration of PTA increases, more acetyl-phosphate can be converted to AcCoA over time, resulting in a decrease in the concentration of the byproduct acetic acid. Therefore, as a method to eliminate the byproduct, AcCoA synthetase (ACS) was added to convert acetic acid to AcCoA. To this end, we used 20 mM glycolaldehyde as a substrate, added an additional 2 mg ml^−1^ ACS under the optimal enzyme conditions as described previously, and conducted the reaction at room temperature. Unfortunately, the addition of ACS resulted in a significant decrease rather than an increase in α-farnesene production (Fig. 4E). This outcome may be caused by the competitive use of CoA by ACS, which prevented PTA from converting acetyl-phosphate to AcCoA in a normal and timely manner; thus, the conversion of acetic acid by ACS was less efficient. Here, 13 μM α-farnesene was obtained in the presence of a 50 mM sodium phosphate solution (pH 7.4) containing 20 mM glycolaldehyde, 1 mM CoA, 5 mM ATP, 1 mM NADPH, 30 mM KCl, 1 mM TCEP, 10 mM MgCl_2_, 3.5 mg ml^−1^ ACPS, 0.5 mg ml^−1^ PTA, and PhaA/Hmgs/Hmgr/Mvk/Pmvk/Mdc/IDI/ISPA/AFS at a molar ratio of 1:10:2:5:5:2:5:2:2. The reaction was performed at room temperature. The effect of glycolaldehyde concentrations ranging from 10 to 30 mM on the yield of α-farnesene was further monitored. The yield of α-farnesene increased with increasing concentrations of glycolaldehyde, reaching 16.4 μM α-farnesene produced from 30 mM glycolaldehyde (Fig. 4F).

### Synthesis of α-farnesene from glycolaldehyde with TMs

**Fig. 5.**
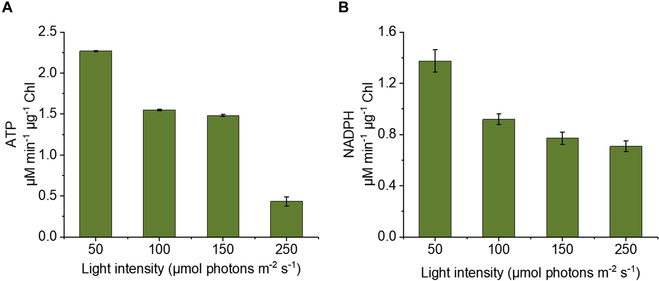
Light-driven cofactor regeneration by TMs under different light intensities. (A) ATP production. (B) NADPH production.

Among the pathways for the synthesis of α-farnesene from methanol, the MVA pathway consumes 3 ATP molecules and 2 NADPH molecules. Since ATP and NADPH are costly and cannot be self-generated in vitro, the failure to incorporate cofactor regeneration could result in an even more expensive synthetic pathway. To this end, we proposed the use of TMs to regenerate NAPDH and ATP in module 2. The integrity and light-harvesting ability of the TMs isolated from *S. oleracea* were verified through large-pore blue native polyacrylamide gel electrophoresis (lpBN-PAGE) (Fig. S10A) and steady-state absorption spectroscopy, with the absorbance ranging from 350 to 750 nm (Fig. S10B). Nine bands, including the PSI-PSII megacomplex, PSI supercomplex, PSII core dimer, ATPase, Cyt*_b6f_* supercomplex, PSII core monomer less CP43, LHCII trimer, and LHCII monomer, were consistent with the information described by Timperio et al. [39] The Chl*a/b* ratio of 2.8 was calculated according to the absorbances at 470 and 650 nm corresponding to Chl*b*, and the bands at 440 and 680 nm corresponded to Chl*a*. The band at 485 nm is attributed to the electron transitions of carotenoids. The ATP and NADPH regeneration rates were also determined. At 50 μmol photons m^−2^ s^−1^, the maximal reduction rate of NADP^+^ to NADPH was 1.38 μM min^−1^ μg^−1^ Chl, and the maximum production rate of ATP was 2.27 μM min^−1^ μg^−1^ Chl. The ratio of ATP/NADPH was approximately 2:1, which is in accordance with the results of Erb and colleagues [16]. However, the regeneration rates of NADPH and ATP decreased with increasing light intensity (Fig. 5A and B), possibly due to the formation of reactive oxygen species, which could damage components of the photosynthetic apparatus, such as the D1 subunit [40]. These results confirmed the intact structure, light-harvesting function, and photoreactivity of the TMs in vitro.

**Fig. 6.**
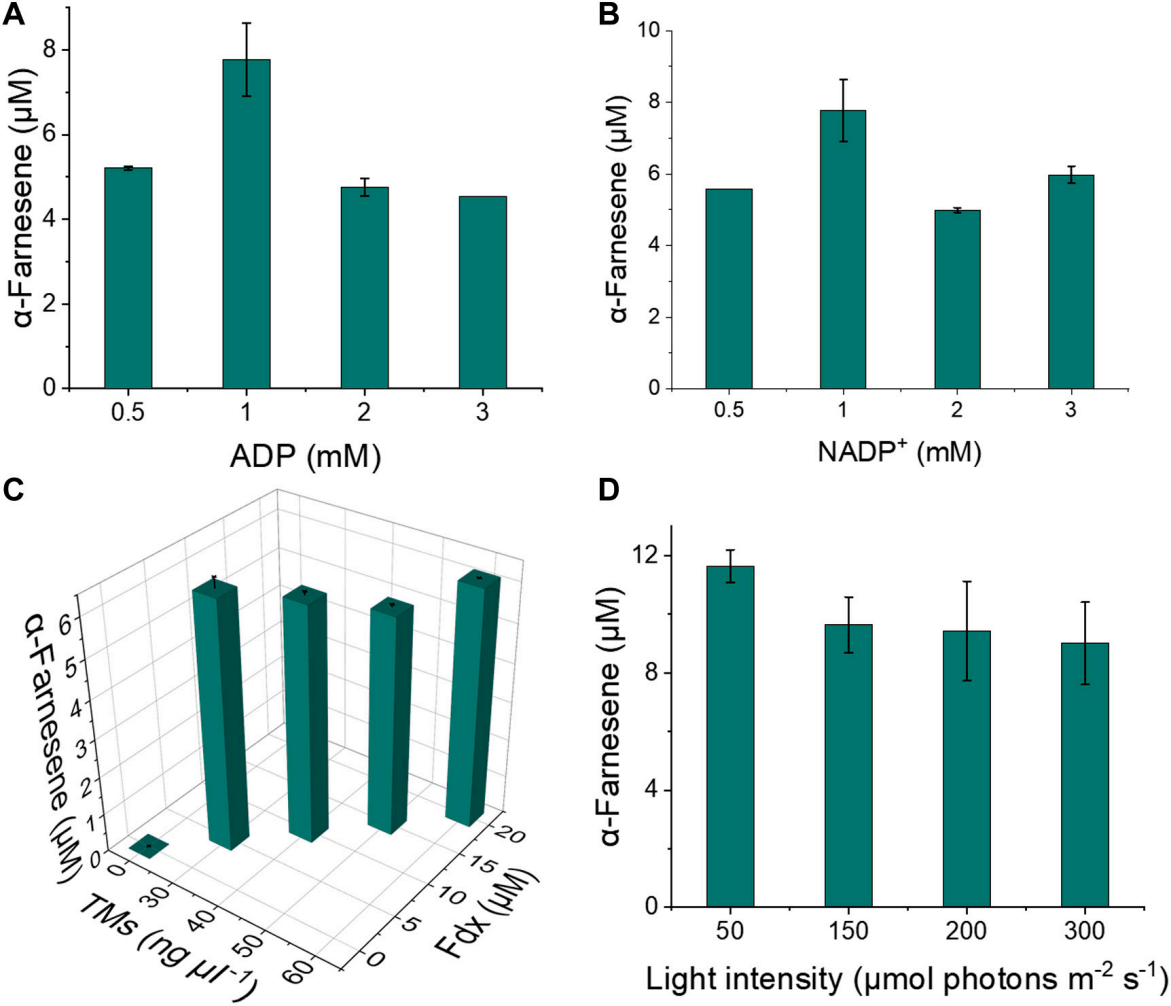
Optimization of module 2 driven by light-powered TMs. (A) Optimization of the ADP concentration. (B) Optimization of NADP^+^ concentration. (C) Optimization of the TM and Fdx concentrations. The optimizations based on the reaction system at room temperature with 50 μmol photons m^−2^ s^−1^ of illumination included a 50 mM sodium phosphate solution (pH 7.4) containing 20 mM glycolaldehyde, 1 mM CoA, 30 mM KCl, 0.5 M betaine, 10 mM sodium L-ascorbate, 1 mM TCEP, 10 mM MgCl_2_, 3.5 mg ml^−1^ ACPS, 0.5 mg ml^−1^ PTA, and PhaA/Hmgs/Hmgr/Mvk/Pmvk/Mdc/IDI/ISPA/AFS at a molar ratio of 1:10:2:5:5:2:5:2:2. (D) Effect of the light intensity on the yield of α-farnesene.

Light-driven cofactor regeneration was tested using the above optimized conditions, and cofactors such as ADP and NADP^+^ were optimized to prove the feasibility of our design. The ADP concentration was first optimized in a 50 mM sodium phosphate solution (pH 7.4) containing 20 mM glycolaldehyde, 1 mM CoA, 0.5 to 3 mM ADP, 1 mM NADP^+^, 30 mM KCl, 0.5 M betaine, 10 mM sodium L-ascorbate, 1 mM TCEP, 10 mM MgCl_2_, 3.5 mg ml^−1^ ACPS, 0.5 mg ml^−1^ PTA, 5 μM Fdx, 10 μg TMs, and PhaA/Hmgs/Hmgr/Mvk/Pmvk/Mdc/IDI/ISPA/AFS at a molar ratio of 1:10:2:5:5:2:5:2:2. The reaction was performed at room temperature with 50 μmol photons m^−2^ s^−1^ of illumination. The greatest amount of α-farnesene synthesized reached 7.8 μM when the ADP concentration was 1 mM (Fig. 6A). Next, the concentration of NADP^+^ was optimized based on the ADP concentration at 1 mM, providing an optimal concentration of NADP^+^ of 1 mM (Fig. 6B). The addition of external Fdx has been reported to be indispensable; thus, the TMs and Fdx concentrations were optimized, and 30 μg ml^−1^ TMs with 5 μM Fdx were suggested as the optimal conditions (Fig. 6C). Finally, we investigated the effect of different light intensities on the yield of α-farnesene. The light intensity was varied from 50 to 300 μmol photons m^−2^ s^−1^, while the other conditions were the same. The experimental results showed that the highest yield of farnesene was obtained at a light intensity of 50 μmol photons m^−2^ s^−1^ (Fig. 6D). As the rate of ATP and NADPH regeneration decreased with increasing light intensity, α-farnesene production also decreased. These results indicated the feasibility of our biosystem for the in vitro synthesis of α-farnesene from glycolaldehyde driven by light-powered TMs.

**Fig. 7.**
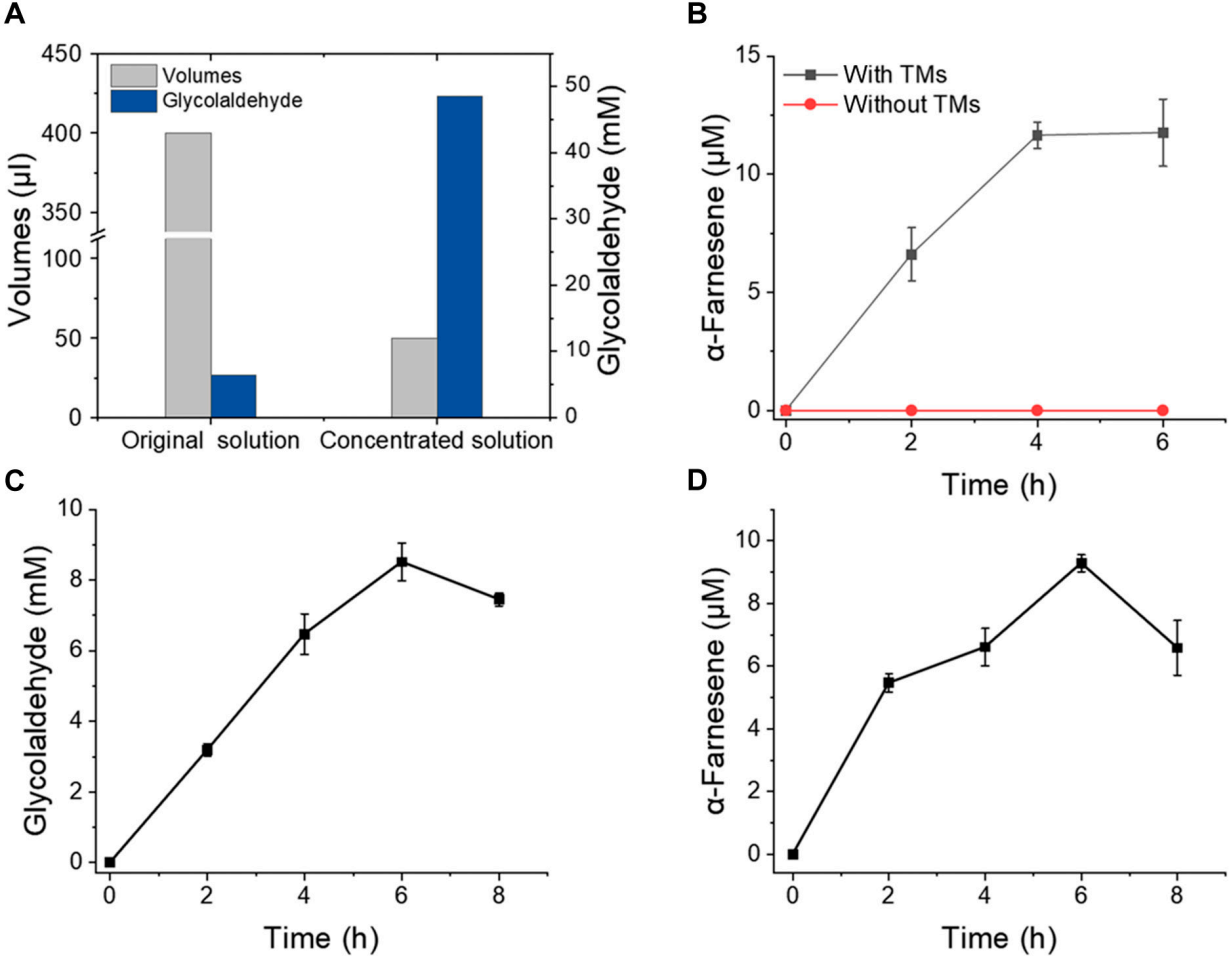
Coupling of module 1 to module 2. (A) Glycolaldehyde from module 1 concentrated by a vacuum concentrator. (B) Synthesis of α-farnesene in the biosystem with and without the addition of TMs using 20 mM glycolaldehyde obtained from module 1 as the substrate. (C) Glycolaldehyde concentration in a 10-ml reaction. (D) α-Farnesene concentration in a 10-ml reaction.

Finally, we attempted to couple the 2 modules for the synthesis of α-farnesene from methanol. However, we failed to synthesize α-farnesene from methanol in one pot. This result could be caused by the toxicity of methanol or the intermediate formaldehyde, which hinders the stable functioning of some enzymes in the pathway. Therefore, we proposed conducting the 2 modules individually and integrating a vacuum concentration process between module 1 and module 2 to switch the reaction buffer and increase the glycolaldehyde concentration, which ensured the successful operation of module 2. In module 1, glycolaldehyde was synthesized in a 100 mM Hepes solution containing 5 mM MgSO_4_ and 1 mM ThDP. Methanol (30 mM) was used as the substrate, along with 0.7 mg ml^−1^ AOX, 0.4 mg ml^−1^ Cat, and 2 mg ml^−1^ *Ec*GCL. After 4 h, the reaction was terminated with acetonitrile due to its high volatility. After centrifugation at 13,000 rpm for 10 min to remove the protein, the solution was concentrated in a vacuum concentrator at 40°C. The solution volume decreased from 400 to 50 μl, resulting in an increase in the glycolaldehyde concentration to 48.5 mM (Fig. 7A). The concentration of glycolaldehyde decreased by approximately 4.2%. Subsequently, the concentration of glycolaldehyde was adjusted to 20 mM, and glycolaldehyde was used as the substrate for module 2. Reactions with or without TMs were assayed under illumination with 50 μmol photons m^−2^ s^−1^ to confirm that the α-farnesene synthetic biosystem was driven by light-powered TMs. As shown in Fig. 7B, only reactions with TMs and under illumination can produce approximately 11.8 μM (2.40 mg liter^−1^) α-farnesene in 6 h, corresponding to a molar conversion efficiency of 0.54% based on glycolaldehyde. Considering a 77% conversion yield from methanol to glycolaldehyde, 7 μM α-farnesene (1.43 mg liter^−1^) could be obtained from methanol, corresponding to a 0.42% molar conversion efficiency. Compared with the optimized results for the individual modules above, the efficiency of the TMs in converting ADP and NADP^+^ to ATP and NADPH was still much lower than that from the direct addition of ATP and NADPH. The low conversion efficiency may have resulted from an insufficient supply of coenzymes and the incompatibility of some enzymes in the pathway. Furthermore, the reaction volumes of modules 1 and 2 increased from 0.5 to 10 ml, while the concentrations of all the components remained unchanged. The molar conversion efficiency of methanol to glycolaldehyde was reduced to 57%, while the yield of α-farnesene was slightly reduced to 9 μM (Fig. 7C and D), suggesting the good scale-up potential of this biosystem.

Regardless, compared to other studies, the yield of α-farnesene synthesized from methanol by our biosystem can still be improved. The low yield of α-farnesene is attributed to the instability and incompatibility of the enzymes and TMs. In the future, the yield and productivity of α-farnesene can be improved by modifying the enzymes in the pathway to increase their activity, selectivity, and stability. Overcoming the rate-limiting step in this pathway is very important, particularly to increase the supply of AcCoA and further increase the regeneration of cofactors. Additionally, the immobilization of enzymes and TMs on certain matrices, such as metal organic frameworks, silica gel, and hydrogels, would result in high stability, activity, and reusability.

## Conclusion

In summary, a light-powered in vitro enzymatic biosystem for α-farnesene synthesis with methanol as the C1 substrate was constructed. Although the α-farnesene yield was not high enough, this work revealed the feasibility of terpene synthesis from C1 compounds by an in vitro light-powered enzymatic biosystem. Our study also suggests a promising direction for future C1 utilization and may guide the engineering of photosynthetic cell factories for the synthesis of many other terpenes.

## Data Availability

The data are available from the authors upon a reasonable request.

## Supplementary Materials

Supplementary 1 Figs. S1 to S10 Tables S1 to S5 References

## References

[1] Bution ML, Molina G, Abrahão MRE, Pastore GM. Genetic and metabolic engineering of microorganisms for the development of new flavor compounds from terpenic substrates. Crit Rev Biotechnol. 2014;35(3):313–325. 10.3109/07388551.2013.855161 24494701

[2] Li C, Zha W, Li W, Wang J, You A. Advances in the biosynthesis of terpenoids and their ecological functions in plant resistance. Int J Mol Sci. 2023;24(14):11561–11577. 37511319 10.3390/ijms241411561 PMC10380271

[3] Jaeger R, Cuny E. Terpenoids with special pharmacological significance: A review. Nat Prod Commun. 2016;11(9):1373–1390. 30807045

[4] Jiang F, Gong T, Chen J, Chen T, Yang J, Zhu P. Synthetic biology of plants-derived medicinal natural product. Chin J Biotechnol. 2021;37(6):1931–1951. 10.13345/j.cjb.210138 34227286

[5] Lee HJ, Lee J, Lee SM, Um Y, Kim Y, Sim SJ, Ji C, Woo HM. Direct conversion of CO_2_ to *α*-farnesene using metabolically engineered *Synechococcus elongatus* PCC 7942. J Agric Food Chem. 2017;65(48):10424–10428. 29068210 10.1021/acs.jafc.7b03625

[6] Cheng T, Wang L, Sun C, Xie C. Optimizing the downstream MVA pathway using a combination optimization strategy to increase lycopene yield in *Escherichia coli*. Microb Cell Factories. 2022;21(1):121–133. 10.1186/s12934-022-01843-z PMC9208136 35718767

[7] Wang J, Jiang W, Liang C, Zhu L, Li Y, Mo Q, Xu S, Chu A, Zhang L, Ding Z, et al. Overproduction of *α*-farnesene in *Saccharomyces cerevisia*e by farnesene synthase screening and metabolic engineering. J Agric Food Chem. 2021;69(10):3103–3113. 33683134 10.1021/acs.jafc.1c00008

[8] Yang L, Gong Q, Lv J, Zhou B, Li G, Guo J. Opportunities and challenges of in vitro synthetic biosystem for terpenoids production. Biotechnol Bioprocess Eng. 2022;27(5):697–705.

[9] Dirkmann M, Nowack J, Schulz F. An in vitro biosynthesis of sesquiterpenes starting from acetic acid. Chembiochem. 2018;19(20):2146–2151. 30085399 10.1002/cbic.201800128

[10] Erdene UB, Billingsley JM, Turner WC, Lichman BR, Ippoliti FM, Garg NK, O’Connor SE, Tang Y. Cell-free total biosynthesis of plant terpene natural products using an orthogonal cofactor regeneration system. ACS Catal. 2021;11(15):9898–9903. 35355836 10.1021/acscatal.1c02267 PMC8963176

[11] Korman TP, Opgenorth PH, Bowie JU. A synthetic biochemistry platform for cell free production of monoterpenes from glucose. Nat Commun. 2017;8:15526. 28537253 10.1038/ncomms15526 PMC5458089

[12] Zhang YHPJ, Zhu Z, You C, Zhang L, Liu K. *In vitro* biotransformation (ivBT): Definitions, opportunities, and challenges. Synth Biol Eng. 2023;1(2):1–37.

[13] Shi T, Han P, You C, Zhang PJ. An in vitro synthetic biology platform for emerging industrial biomanufacturing: Bottom-up pathway design*. Synth Syst*. Biotechnol. 2018;3(3):186–195. 10.1016/j.synbio.2018.05.002 PMC6190512 30345404

[14] Wang W, Yang J, Sun Y, Li Z, You C. Artificial ATP-free in vitro synthetic enzymatic biosystems facilitate aldolase-mediated C–C bond formation for biomanufacturing. ACS Catal. 2019;10(2):1264–1271.

[15] Wang Y, Huang W, Sathitsuksanoh N, Zhu Z, Zhang YHP. Biohydrogenation from biomass sugar mediated by in vitro synthetic enzymatic pathways. Chem Biol. 2011;18(3):372–380. 21439482 10.1016/j.chembiol.2010.12.019

[16] Mille TE, Beneyton T, Schwander T, Diehl C, Girault M, McLean R, Chotel T, Claus P, Cortina NS, Baret JC, et al. Light-powered CO_2_ fixation in a chloroplast mimic with natural and synthetic parts. Science. 2020;368(6491):649–654. 32381722 10.1126/science.aaz6802 PMC7610767

[17] Li F, Wei X, Zhang L, Liu C, You C, Zhu Z. Installing a green engine to drive an enzyme cascade: A light-powered in vitro biosystem for poly(3-hydroxybutyrate) synthesis. Angew Chem Int Ed Engl. 2022;61(1): Article e202111054. 34664348 10.1002/anie.202111054

[18] Jiang W, Villamor DH, Peng H, Chen J, Liu L, Haritos V, Amaro RL. Metabolic engineering strategies to enable microbial utilization of C1 feedstocks. Nat Chem Biol. 2021;17(8):845–855. 34312558 10.1038/s41589-021-00836-0

[19] Qiao Y, Ma W, Zhang S, Guo F, Liu K, Jiang Y, Wang Y, Xin F, Zhang W, Jiang M. Artificial multi-enzyme cascades and whole-cell transformation for bioconversion of C1 compounds: Advances, challenge and perspectives*. Synth Syst*. Biotechnol. 2023;8(4):578–583. 10.1016/j.synbio.2023.08.008 PMC10495606 37706206

[20] Bogorad IW, Chen CT, Theisen MK, Wu TY, Schlenz AR, Lam AT, Liao JC. Building carbon–carbon bonds using a biocatalytic methanol condensation cycle. Proc Natl Acad Sci USA. 2014;111(45):15928–15933. 25355907 10.1073/pnas.1413470111 PMC4234558

[21] Güner S, Wegat V, Pick A, Sieber V. Design of a synthetic enzyme cascade for the in vitro fixation of a C1 carbon source to a functional C4 sugar. Green Chem. 2021;23(17):6583–6590.

[22] Cai T, Sun H, Qiao J, Zhu L, Zhang F, Zhang J, Tang Z, Wei X, Yang J, Yuan Q, et al. Cell-free chemoenzymatic starch synthesis from carbon dioxide. Science. 2021;373(6562):1523–1527. 34554807 10.1126/science.abh4049

[23] Lu X, Liu Y, Yang Y, Wang S, Wang Q, Wang X, Yan Z, Cheng J, Liu C, Yang X, et al. Constructing a synthetic pathway for acetyl-coenzyme a from one-carbon through enzyme design. Nat Commun. 2019;10(1):1378–1388. 30914637 10.1038/s41467-019-09095-z PMC6435721

[24] Zhang J, Liu D, Liu Y, Chu H, Cheng J, Zhao H, Fu S, Huihong L, Fu Y, Ma Y, et al. Hybrid synthesis of bioplastics polyhydroxybutyrate from carbon dioxide. Green Chem. 2023;25(8):3247–3255.

[25] Ak B, Glasemacher J, Schmidt R, Schönheit P. Purification and characterization of two extremely thermostable enzymes, phosphate acetyltransferase and acetate kinase, from the hyperthermophilic eubacterium *Thermotoga maritima*. J Bacteriol. 1999;181(6):1861–1867. 10074080 10.1128/jb.181.6.1861-1867.1999 PMC93586

[26] Strand DD, Fisher N, Kramer DM. The higher plant plastid NAD(P)H dehydrogenase-like complex (NDH) is a high efficiency proton pump that increases ATP production by cyclic electron flow. J Biol Chem. 2017;292(28):11850–11860. 28559282 10.1074/jbc.M116.770792 PMC5512078

[27] Berny DS, Salvi D, Joyard J, Rolland N. Purification of intact chloroplasts from Arabidopsis and spinach leaves by isopycnic centrifugation. Curr Protoc Cell Biol. Chapter 3:2008, Unit 3 30. 10.1002/0471143030.cb0330s40 18819091

[28] Shi T, Liu S, Zhang YPJ. CO_2_ fixation for malate synthesis energized by starch via in vitro metabolic engineering. Metab Eng. 2019;55:152–160. 31306776 10.1016/j.ymben.2019.07.005

[29] Chen H, Zhang YPJ. Enzymatic regeneration and conservation of ATP: Challenges and opportunities. Crit Rev Biotechnol. 2021;41(1):16–33. 33012193 10.1080/07388551.2020.1826403

[30] Honda K, Hara N, Cheng M, Nakamura A, Mandai K, Okano K, Ohtake H. *In vitro* metabolic engineering for the salvage synthesis of NAD^+^. Metab Eng. 2016;35:114–120. 26912312 10.1016/j.ymben.2016.02.005

[31] Rayder TM, Adillon EH, Byers JA, Tsung CK. A bioinspired multicomponent catalytic system for converting carbon dioxide into methanol autocatalytically. Chem. 2020;6(7):1742–1754.

[32] Kim JH, Cheon H, Jo HJ, Kim JW, Kim GY, Seo HR, Seo PW, Kim JS, Park JB. Engineering of two thiamine diphosphate-dependent enzymes for the regioselective condensation of C1-formaldehyde into C4-erythrulose. Int J Biol Macromol. 2023;253(8): Article 127674. 37890751 10.1016/j.ijbiomac.2023.127674

[33] Pechous SW, Whitaker BD. Cloning and functional expression of an ( E , E )-a-farnesene synthase cDNA from peel tissue of apple fruit. Planta. 2004;219(1):84–94. 14740213 10.1007/s00425-003-1191-4

[34] Frey S, Görlich D. A new set of highly efficient, tag-cleaving proteases for purifying recombinant proteins. J Chromatogr A. 2014;1337:95–105. 24636565 10.1016/j.chroma.2014.02.029

[35] Goldsmith M, Barad S, Peleg Y, Albeck S, Dym O, Brandis A, Mehlman T, Reich Z. The identification and characterization of an oxalyl-CoA synthetase from grass pea (*Lathyrus sativus* L.)*. RSC*. Chem Biol. 2022;3(3):320–333. 10.1039/d1cb00202c PMC8905533 35359497

[36] Mamipour M, Yousefi M, Hasanzadeh M. An overview on molecular chaperones enhancing solubility of expressed recombinant proteins with correct folding. Int J Biol Macromol. 2017;102:367–375. 28412337 10.1016/j.ijbiomac.2017.04.025 PMC7185796

[37] Zhu F, Zhong X, Hu M, Lu L, Deng Z, Liu T. *In vitro* reconstitution of mevalonate pathway and targeted engineering of farnesene overproduction in *Escherichia coli*. Biotechnol Bioeng. 2014;111(7):1396–1405. 24473754 10.1002/bit.25198

[38] Movahedi A, Wei H, Pucker B, Zefrehei RF, Ghaderi M, Kiani-Pouya A, Jiang T, Zhuge Q, Yang L, et al. Isoprenoid biosynthesis regulation in poplars by methylerythritol phosphate and mevalonic acid pathways*. Front*. Plant Sci. 2022;13: Article 968780. 10.3389/fpls.2022.968780 PMC9562105 36247639

[39] Timperio AM, Amici GMDQ, Barta C, Loreto F, Zolla L. Proteomics, pigment composition, and organization of thylakoid membranes in iron-deficient spinach leaves. J Exp Bot. 2007;58(13):3695–37103695–3710. 17928371 10.1093/jxb/erm219

[40] Andersson B, Aro E-M. Photodamage and D1 protein turnover in photosystem II. In: Aro E-M, Andersson B, editors. *Regulation of photosynthesis*. Dordrecht: Springer; 2001. p. 373–393.

