# Supplementary material for: A Light-Driven In Vitro Enzymatic Biosystem for the Synthesis of α-Farnesene from Methanol
Source: Biodes Res. 2024 Jul 30;6:0039. doi: 10.34133/bdr.0039 (PMC11286291; doi:10.34133/bdr.0039)
Supplement: Supplementary 1 — Figs. S1 to S10 Tables S1 to S5 References [file bdr.0039.f1.docx]

**Supplementary Materials**

**A light-driven *in vitro* enzymatic biosystem for α-farnesene synthesis from methanol**

Xinyue Gui^1,2†^, Fei Li^2†^, Xinyu Cui^2,3^, Ranran Wu^2^, Dingyu Liu^2^, Chunling Ma^2^, Lijuan Ma^1^, Huifeng Jiang^2,3^, Chun You^3*^, Zhiguang Zhu^2,3*^

^1^ Key Laboratory of Industrial Fermentation Microbiology, Ministry of Education, Tianjin Key Laboratory of Industrial Microbiology, The College of Biotechnology, Tianjin University of Science and Technology, Tianjin, 300457, China

^2^ Key Laboratory of Engineering Biology for Low-Carbon Manufacturing, Tianjin Institute of Industrial Biotechnology, Chinese Academy of Sciences, Tianjin, 300308, China

^3^ University of Chinese Academy of Sciences, Beijing, 100049, China

*Address correspondence to: [zhu_zg@tib.cas.cn](mailto:zhu_zg@tib.cas.cn), you_c@tib.cas.cn

†These authors contributed equally to this work.

**Supporting Figures and Tables**


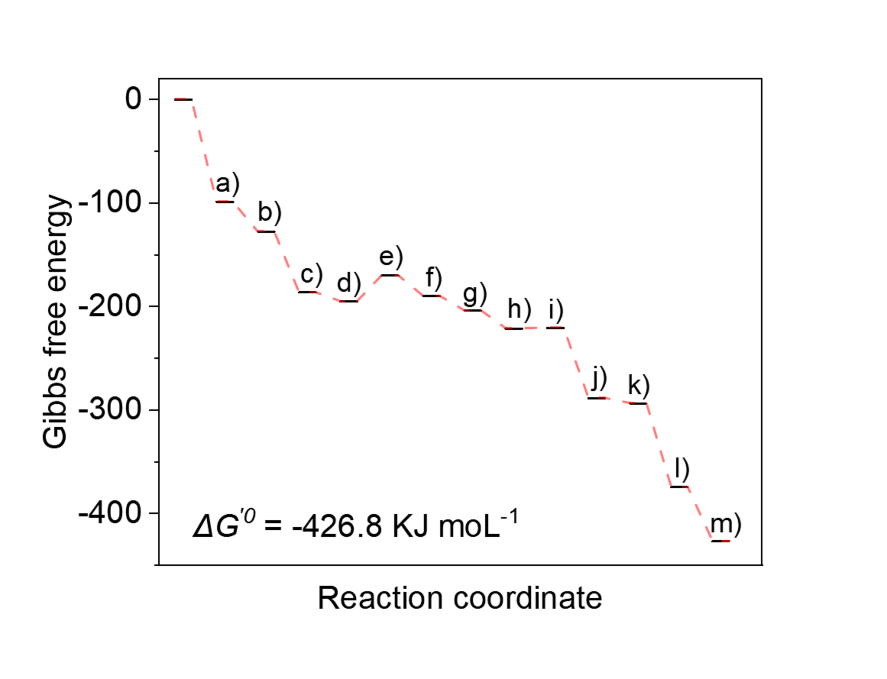


**Fig. S1.** Standard Gibbs free energy change of each reaction step and the overall process at pH 7.5 and an ionic strength of 0.25 M. The Gibbs free energy change of each reaction labeled a), b), c), d), e), f), g), h), i), j), k), l), m) was shown according to Table S2. The overall Δ*G^’0^* was calculated to be -426.8 kJ moL^-1^.


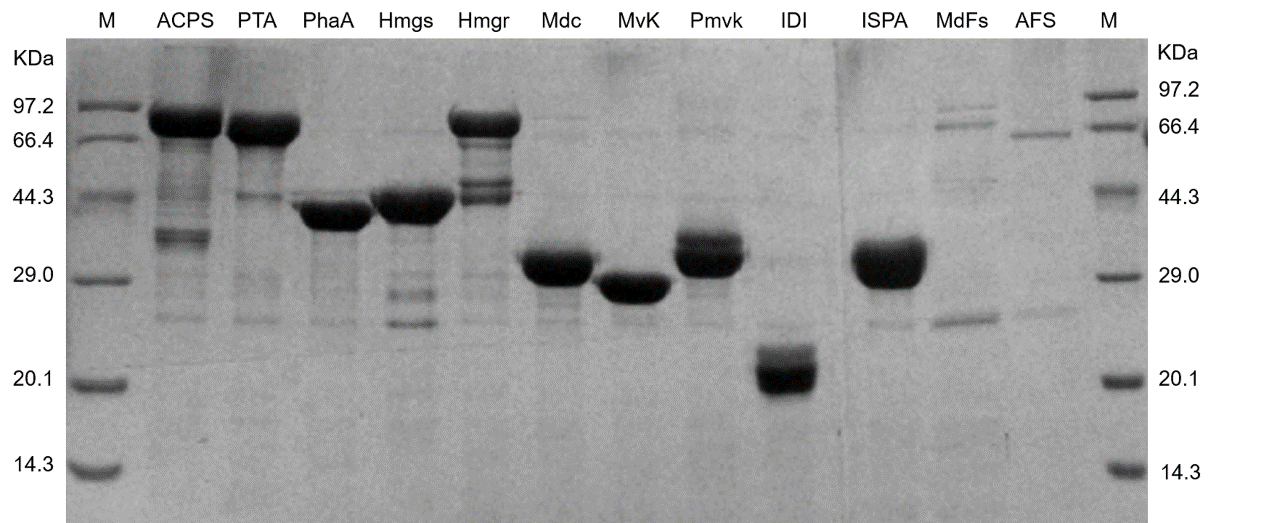


**Fig. S2.** Preparation of enzymes. SDS-PAGE analysis of the recombinant enzymes. ACPS: Acetyl-phosphate synthase; PTA: Phosphate acetyltransferase; PhaA: Acetyl-CoA acetyltransferase; Hmgs: HMG-CoA synthase; Hmgr: HMG-CoA reductase; Mdc: Mevalonate-PP decarboxylase; Mvk: Mevalonate kinase; Pmvk: Phosphomevalonate kinase; IDI: Isopentenyl-PP isomerase; ISPA: Farnesyl-PP synthase; MdFs/AFS: α-Farnesene synthase.


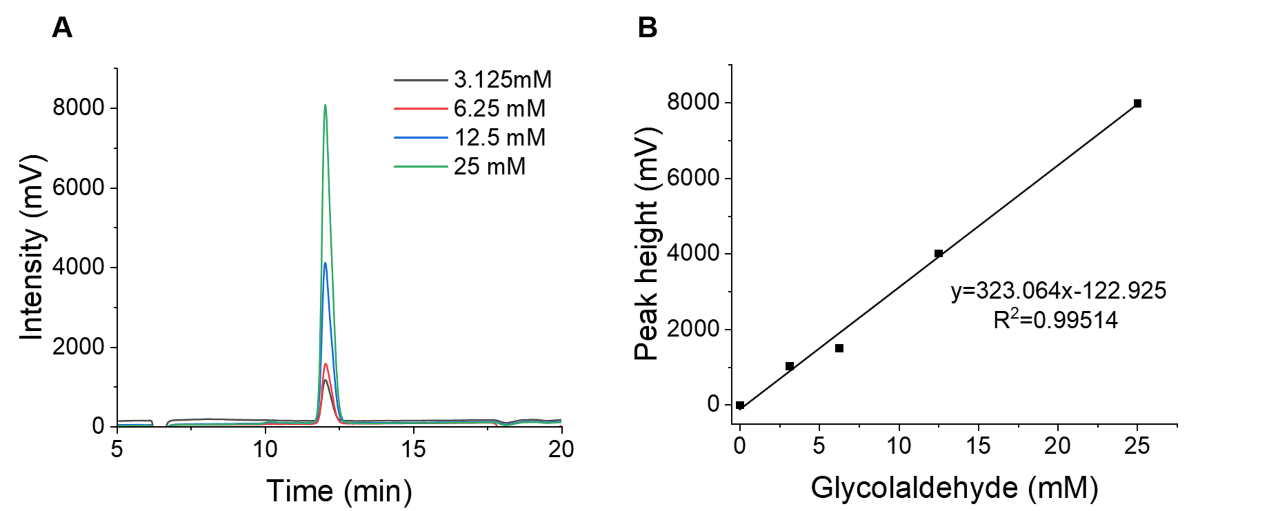


**Fig. S3.** Standard curve of glycolaldehyde set by HPLC. (A) HPLC of glycolaldehyde at concentrations of 3.125 mM, 6.25 mM, 12.5 mM, 25 mM, the retention time was observed at 12 min. (B) Linear regression curve of glycolaldehyde.


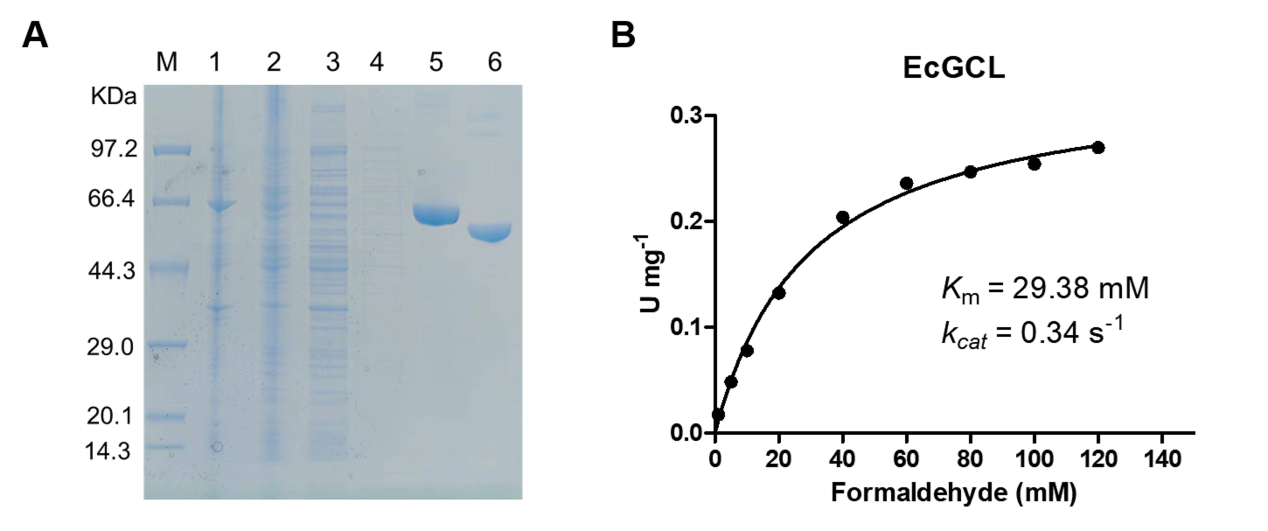


**Fig. S4.** Preparation of *Ec*GCL. (A). SDS-PAGE analysis *Ec*GCL. M: Maker; Lane 1: Broken cells; Lane 2: Supernatant; Lane 3: Precipitate; Lane 4: Flow-through; Lane 5: Purified *Ec*GCL; Lane 6: Purified GALS. (B) Michaelis-Menten curve of *Ec*GCL. Enzyme kinetics were determined with 0.1 mg mL^-1^ *Ec*GCL, the concentration of formaldehyde ranged from 2.5 to 120 mM.


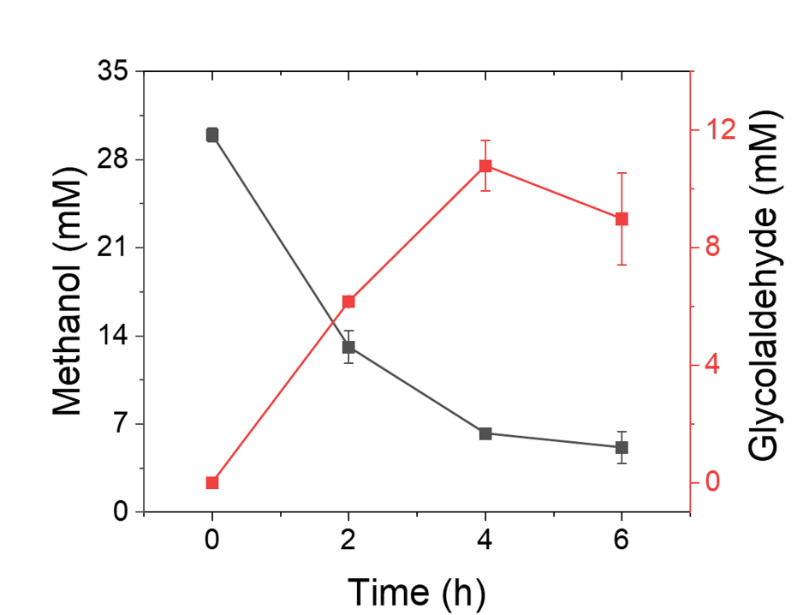


**Fig. S5.** Time profile of glycolaldehyde yield and methanol consumption under optimised conditions. The experiment was performed using 30 mM methanol, 0.7 mg mL^-1^ AOX, 0.4 mg mL^-1^ Cat, and 2 mg mL^-1^ *Ec*GCL in a 100 mM HEPES solution containing 5 mM MgSO_4_ and 1 mM ThDP.


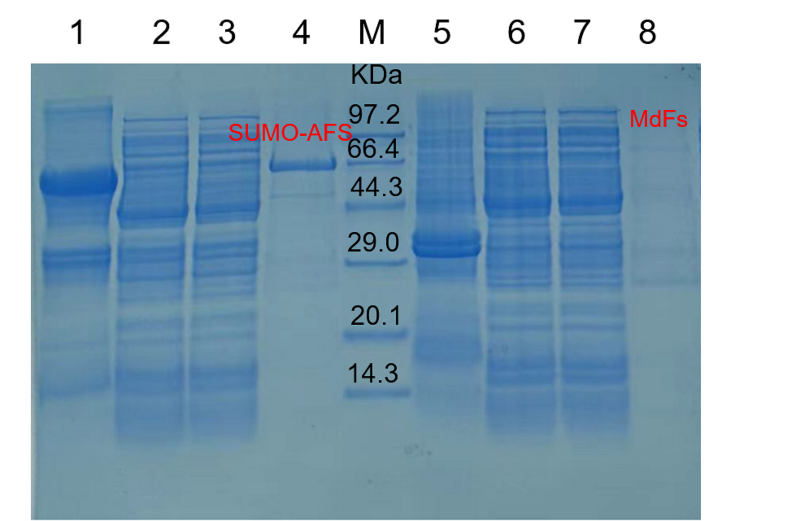


**Fig. S6.** Expression and purification of SUMO-AFS and MdFs. Lane 1: Precipitate of broken cells of AFS; Lane 2: Supernatant of broken cells of AFS; Lane 3: Flow-through of broken cells of AFS; Lane 4: Purified AFS; Lane 5: Precipitate of broken cells of MdFs; Lane 6: Supernatant of broken cells of MdFs; Lane 7: Flow-through of broken cells of MdFs; Lane 8: Purified MdFs.


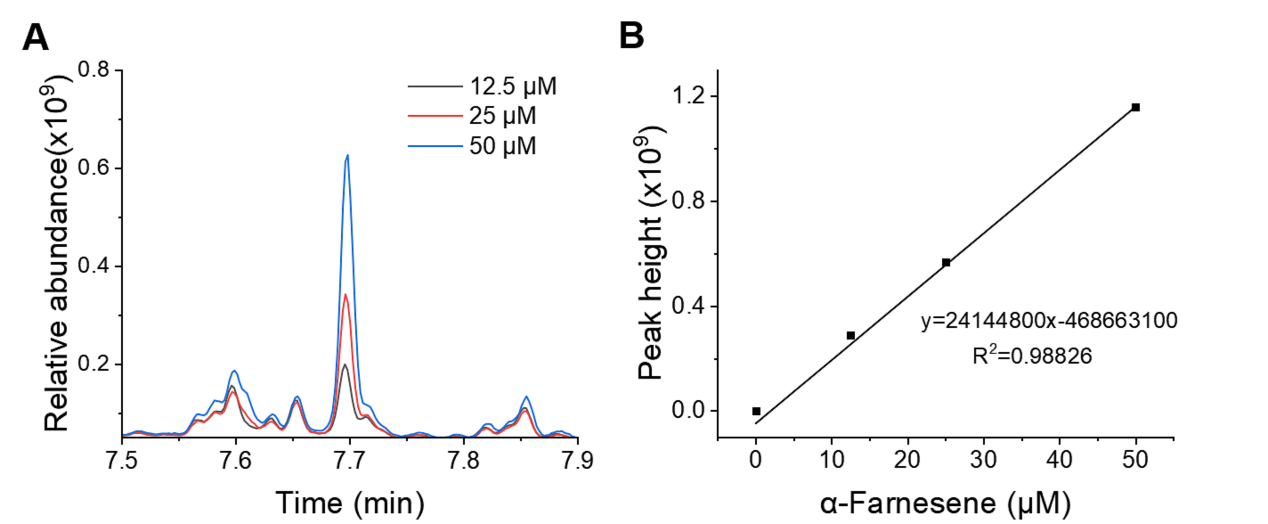


**Fig. S7.** Standard curve of α-farnesene set by GC-MS. (A) GC graph of α-farnesene at concentrations of 12.5 μM, 25 μM, 50 μM, the retention time was observed at 7.7 min. (B) Linear regression curve of α-farnesene.


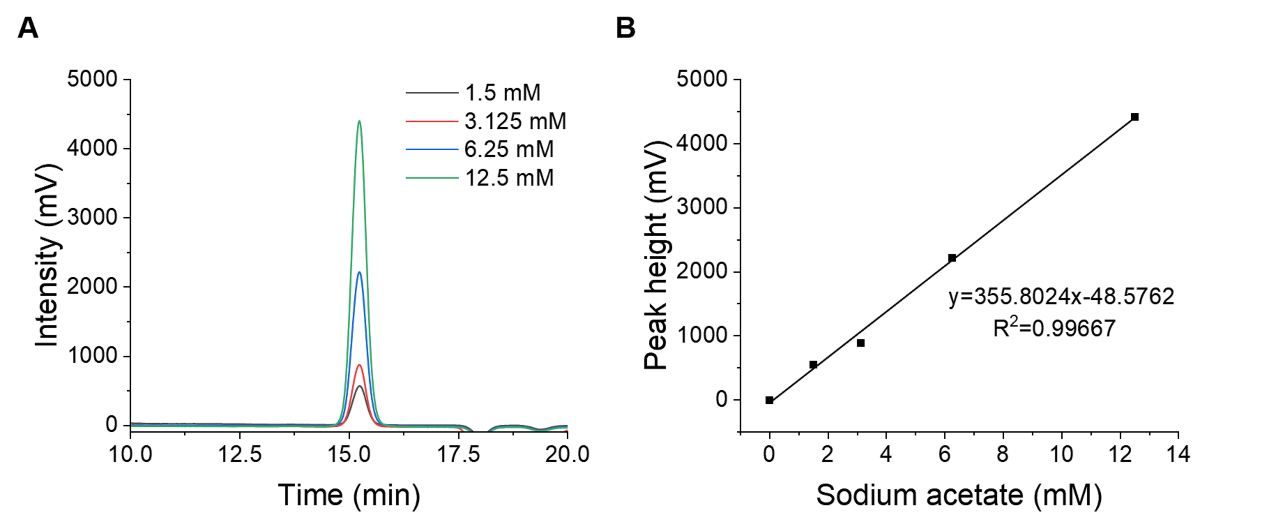


**Fig. S8.** HPLC chromatogram and standard curve of sodium acetate. (A) HPLC of sodium acetate at concentrations of 1.5 μM, 3.125 μM, 6.25 μM, 12.5 μM, the retention time was observed at 15.2 min. (B) Standard curve of sodium acetate was build-up by HPLC equipped with Aminex HPX-87H column and the HPLC conditions set at 40°C with a mobile phase of 5 mM sulphuric acid and a flow rate of 0.6 mL min^-1^.


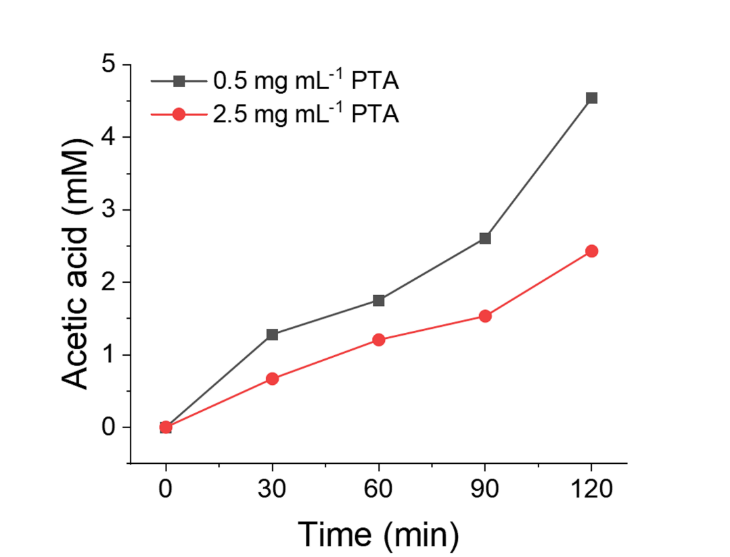


**Fig. S9.** Acetic acid produced in the pathway. Acetic acid production at different enzyme levels under the condition of 50 mM sodium phosphate buffer (pH 7.4) containing 30 mM glycolaldehyde 5 mM MgCl_2_, 10 mM KCl, 2.5 mg mL^-1^ ACPS, 1 mM CoA, 1 mM ThDP at room temperature. Concentration of PTA varied from 0.5 to 2.5 mg mL^-1^.


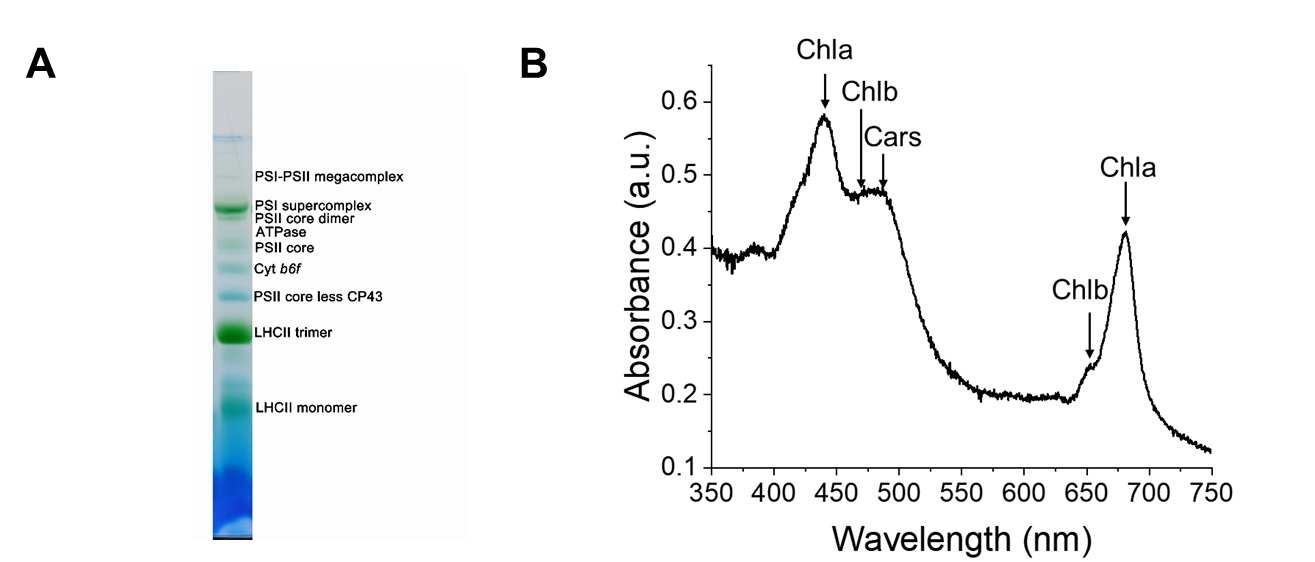


**Fig. S10.** Characteristics of thylakoid membranes. (A) lpBN-PAGE analysis of photosynthetic apparatus isolated from Spinach. 7 μg TMs (equivalent of chlorophylls) was loaded. PSI: photosystem I; PSII: photosystem II; Cyt*_b6f_*: cytochrome *b6f* complex; LHCII: light-harvesting complex of photosystem II; CP43: photosystem II chlorophyll binding subunit. (B) Steady-state absorption spectrum of TMs isolated from Spinach. The absorbance at 470 and 650 nm corresponds to Chl*b*, and the bands at 440 and 680 nm correspond to Chl*a*. Band at 485 nm correspond to Cars. Chl*a*: chlorophyll *a*, Chl*b*: chlorophyll *b*, Cars: carotenoids.

**Table S1.** Information of enzymes used in this study

| Enzyme | Full name | Source | EC | Sp.act. (U mg^-1^) | Re. |
| --- | --- | --- | --- | --- | --- |
| AOX | Alcohol oxidase | Sigma-Aldrich |  |  |  |
| Cat | Catalase | Auwitkey |  |  |  |
| GALS | Glycolaldehyde synthase | *E. coli* | 4.1.1.7 | 0.0362 | This work |
| ACPS | Acetyl-phosphate synthase | *Bifidobacterium* | 2.7.8.7 | 0.5761 | This work |
| PTA | Phosphate acetyltransferase | *E. coli* | 2.3.1.8 | 0.40635 | This work |
| PhaA | Acetyl‐CoA acetyltransferase | *C. necator* | 2.3.1.9 | 81.1 ± 5.7 | [1, 2] |
| Hmgs | HMG-CoA synthase | *E. faecalis* | 2.3.3.10 | 1.5 ± 0.1 | [1, 2] |
| Hmgr | HMG-CoA reductase | *E. faecalis* | 1.1.1.34 | 4.2 ± 0.4 | [1, 2] |
| Mvk | Mevalonate kinase | *M. mazei* | 2.7.1.36 | 8.1 ± 0.6 | [1, 2] |
| Pmvk | Phosphomevalonate kinase | *S. pneumoniae* | 2.7.4.2 | 14.8 ± 0.1 | [1, 2] |
| Mdc | Mevalonate-PP decarboxylase | *S. pneumoniae* | 4.1.1.33 | 4.1 ± 0.1 | [1, 2] |
| IDI | Isopentenyl-PP isomerase | *E. coli* | 5.3.3.2 | 2.1 | [3] |
| ISPA | Farnesyl-PP synthase | *E. coli* | 2.5.1.10 | n.d. |  |
| MdFs | alpha-Farnesene synthase | *Malus domestica* | 4.2.3.46 | n.d. |  |
| GCL | Glyoxylate carboligase | *E. coli* | 2.2.1.5 | 0.236 | This work |
| AFS | (E, E)-alpha-farnesene synthase | *Malus domestica subsp. chinensis* | 4.2.3.46 | n.d. |  |

**Table S2.** Reactions and Gibbs free energy

| Serial No. | Enzyme | Reaction | ΔG KJ moL^-1^ |
| --- | --- | --- | --- |
| a | AOX | O_2_ + Methanol => Hydrogen peroxide + Formaldehyde | -98.9 ± 10.3 |
| b | GCL | 2 Formaldehyde => Glycolaldehyde | -28.7 ± 6.4 |
| c | ACPS | Glycolaldehyde + pi => Acetyl phosphate + H_2_O | -58.8 ± 3.8 |
| d | PTA | CoA + Acetyl phosphate => pi + Acetyl-CoA | -8.7 ± 1.2 |
| e | PhaA | 2 Acetyl-CoA => CoA + Acetoacetyl-CoA | 25 ± 1.7 |
| f | Hmgs | H_2_O + Acetyl-CoA + Acetoacetyl-CoA => CoA+ (S)-3-Hydroxy-3-methylglutaryl-CoA | -19.8 ± 4.7 |
| g | Hmgr | 2 NADPH + (S)-3-Hydroxy-3-methylglutaryl-CoA => 2 NADP + CoA + (R)-Mevalonate | -14.1 ± 3.4 |
| h | Mvk | ATP+ (R)-Mevalonate => ADP + (R)-5-Phosphomevalonate | -17.2 ± 4.1 |
| i | Pmvk | ATP + (R)-5-Phosphomevalonate => ADP + (R)-5-Diphosphomevalonate | 0.6 ± 4.1 |
| j | Mdc | ATP + (R)-5-Diphosphomevalonate => ADP + pi + CO_2_ + Isopentenyl diphosphate | -68.0 ± 7.8 |
| k | IDI | Isopentenyl diphosphate => Dimethylallyl diphosphate | -4.8 ± 5.7 |
| l | ISPA | 2 Isopentenyl diphosphate + Dimethylallyl diphosphate => ppi + trans,trans-Farnesyl diphosphate | -81.2 ± 5.8 |
| m | AFS | trans,trans-Farnesyl diphosphate => ppi + alpha-Farnesene | -52.2 ± 5.6 |

**Table S3.** Plasmids used in this study.

| **Plasmids** | **Relevant characteristics** | **Source** |
| --- | --- | --- |
| pET28a-*GALS* | pET28a vector, *Nde*І*-GALS-Xho*I | This study |
| pET28a-*GCL* | pET28a vector, *Nde*І*-GCL-Xho*I | This study |
| pET28a-*ACPS* | pET28a vector, *Nde*І*-ACPS-Xho*I | This study |
| pET28a-*PTA* | pET28a vector, *Nde*І*-PTA-Xho*I | This study |
| pET28a-*PhaA* | pET28a vector, *Nde*І*-PhaA-Xho*I | This study |
| pET28a-*Hmgs* | pET28a vector, *Nde*І*-Hmgs-Xho*I | This study |
| pET28a-*Hmgr* | pET28a vector, *Nde*І*-Hmgr-Xho*I | This study |
| pET28a-*Mvk* | pET28a vector, *Nde*І*-Mvk-Xho*I | This study |
| pET28a-*Pmvk* | pET28a vector, *Nde*І*-Pmvk-Xho*I | This study |
| pET28a-*Mdc* | pET28a vector, *Nde*І*-Mdc-Xho*I | This study |
| pET28a-*IDI* | pET28a vector, *Nde*І*-IDI-Xho*I | This study |
| pET28a-*ISPA* | pET28a vector, *Nde*І*-ISPA-Xho*I | This study |
| pET28a-*MdFs* | pET28a vector, *Nde*І*-MdFs-Xho*I | This study |
| pET28a-*SUMO-AFS* | pET28a vector, *Nde*І*-SUMO-AFS-Xho*I | This study |

**Table S4.** Primers used for cloning in this study

| Enzyme | Primer Sequences |
| --- | --- |
| IDI | IF: 5’-TCATCATCATCATCACAGCAGCGGCCAGACCGAACATGTGATTCTGCTGA-3’  IR: 5’-GGGCTTTGTTAGCAGCCGGATCTCATTTCAGCTGCGTAAACGCGCTCAGG-3’  VF: 5’-CCTGAGCGCGTTTACGCAGCTGAAATGAGATCCGGCTGCTAACAAAGCCC-3’  VR: 5’-TCAGCAGAATCACATGTTCGGTCTGGCCGCTGCTGTGATGATGATGATGA-3’ |
| ISPA | IF: 5’-TCATCATCATCATCACAGCAGCGGCGATTTTCCGCAGCAGCTGGAAGCGT-3’  IR: 5’-GGGCTTTGTTAGCAGCCGGATCTCATTTGTTGCGCTGAATAATATAATCC-3’  VF: 5’-GGATTATATTATTCAGCGCAACAAATGAGATCCGGCTGCTAACAAAGCCC-3’  VR: 5’-ACGCTTCCAGCTGCTGCGGAAAATCGCCGCTGCTGTGATGATGATGATGA-3’ |
| MdFs | IF: 5’-TCATCATCATCATCACAGCAGCGGCGAATTTCGCGTGCATCTGCAAGCGG-3’  IR: 5’-GGGCTTTGTTAGCAGCCGGATCTCAGTTCACCAGCGGCTGAAACAGCAGG-3’  VF: 5’-CCTGCTGTTTCAGCCGCTGGTGAACTGAGATCCGGCTGCTAACAAAGCCC-3’  VR: 5’-CCGCTTGCAGATGCACGCGAAATTCGCCGCTGCTGTGATGATGATGATGA-3’ |

**Table S5.** Synthesized genes in this study

| Enzyme | Sequences |
| --- | --- |
| GALS_F397YC398M_ | ATGGCTTCTGTTCACGGTACCACCTACGAACTGCTGCGTCGTCAGGGTATCGACACCGTTTTCGGTAACCCGGGTTCTAACGAACTGCCGTTCCTGAAAGACTTCCCGGAAGACTTCCGTTACATCCTGGCTCTGCAGGAAGCTTGCGTTGTTGGTATCGCTGACGGTTACGCTCAGGCTTCTCGTAAACCGGCTTTCATCAACCTGCACTCTGCTGCTGGTACCGGTAACGCTATGGGTGCTCTGTCTAACGCTCGTACCTCTCACTCTCCGCTGATCGTTACCGCTGGTCAGCAGACCCGTGCTATGATCGGTGTTGAAGCTGGTGAAACCAACGTTGACGCTGCTAACCTGCCGCGTCCGCTGGTTAAATGGTCTTACGAACCGGCTTCTGCTGCTGAAGTTCCGCACGCTATGTCTCGTGCTATCCACATGGCTTCTATGGCTCCGCAGGGTCCGGTTTACCTGTCTGTTCCGTACGACGACTGGGACAAAGACGCTGACCCGCAGTCTCACCACCTGTTCGACCGTCACGTTTCTTCTTCTGTTCGTCTGAACGACCAGGACCTGGACATCCTGGTTAAAGCTCTGAACTCTGCTTCTAACCCGGCTATCGTTCTGGGTCCGGACGTTGACGCTGCTAACGCTAACGCTGACTGCGTTATGCTGGCTGAACGTCTGAAAGCTCCGGTTTGGGTTGCTCCGTCTGCTCCGCGTTGCCCGTTCCCGACCCGTCACCCGTGCTTCCGTGGTCTGATGCCGGCTGGTATCGCTGCTATCTCTCAGCTGCTGGAAGGTCACGACGTTGTTCTGGTTATCGGTGCTCCGGTTTTCCGTTACGTTTTTTACGACCCGGGTCAGTACCTGAAACCGGGTACCCGTCTGATCTCTGTTACCTGCGACCCGCTGGAAGCTGCTCGTGCTCCGATGGGTGACGCTATCGTTGCTGACATCGGTGCTATGGCTTCTGCTCTGGCTAACCTGGTTGAAGAATCTTCTCGTCAGCTGCCGACCGCTGCTCCGGAACCGGCTAAAGTTGACCAGGACGCTGGTCGTCTGCACCCGGAAACCGTTTTCGACACCCTGAACGACATGGCTCCGGAAAACGCTATCTACCTGAACGAATCTACCTCTACCACCGCTCAGATGTGGCAGCGTCTGAACATGCGTAACCCGGGTTCTTACTACTACATGGCTGCTGGTGGTCTGGGTTTCGCTCTGCCGGCTGCTATCGGTGTTCAGCTGGCTGAACCGGAACGTCAGGTTATCGCTGTTATCGGTGACGGTTCTGCTAACTACTCTATCTCTGCTCTGTGGACCGCTGCTCAGTACAACATCCCGACCATCTTCGTTATCATGAACAACGGTACCTACGGTATGCTGCGTTGGTTCGCTGGTGTTCTGGAAGCTGAAAACGTTCCGGGTCTGGACGTTCCGGGTATCGACTTCCGTGCTCTGGCTAAAGGTTACGGTGTTCAGGCTCTGAAAGCTGACAACCTGGAACAGCTGAAAGGTTCTCTGCAGGAAGCTCTGTCTGCTAAAGGTCCGGTTCTGATCGAAGTTTCTACCGTTTCTCCGGTTAAA |
| *Ec*GCL | ATGGCGAAAATGCGCGCGGTGGATGCGGCGATGTATGTGCTGGAAAAAGAAGGCATTACCACCGCGTTTGGCGTGCCGGGCGCGGCGATTAACCCGTTTTATAGCGCGATGCGCAAACATGGCGGCATTCGCCATATTCTGGCGCGCCATGTGGAAGGCGCGAGCCACATGGCGGAAGGCTATACCCGCGCGACCGCGGGCAACATTGGCGTGTGCCTGGGCACGAGCGGCCCGGCGGGCACCGATATGATTACCGCGCTGTATAGCGCGAGCGCGGATAGCATTCCGATTCTGTGCATTACCGGCCAAGCGCCGCGCGCGCGCCTGCATAAAGAAGATTTTCAAGCGGTGGATATTGAAGCGATTGCGAAACCGGTGAGCAAAATGGCGGTGACCGTGCGCGAAGCGGCGCTGGTGCCGCGCGTGCTGCAGCAAGCGTTTCATCTGATGCGCAGCGGCCGCCCGGGCCCGGTGCTGGTGGATCTGCCGTTTGATGTGCAAGTGGCGGAAATTGAATTTGATCCGGATATGTATGAACCGCTGCCGGTGTATAAACCGGCGGCGAGCCGCATGCAGATTGAAAAAGCGGTGGAAATGCTGATTCAAGCGGAACGCCCGGTGATTGTGGCGGGCGGTGGCGTGATTAACGCGGATGCGGCCGCGCTGCTGCAGCAGTTTGCGGAACTGACGAGCGTGCCGGTGATTCCGACCCTGATGGGCTGGGGCTGCATTCCGGATGATCATGAACTGATGGCGGGCATGGTGGGCCTGCAGACCGCGCATCGCTATGGCAACGCGACCCTGCTGGAAAGCGATATGGTGTTTGGCATTGGCAACCGCTTTGCGCAGAAACATACCGGCAGCGTGGAAAAATATACCGAAGGCCGCAAAATTGTGCATATTGATATTGAACCGACGCAGATTGGCCGCGTGCTGTGCCCGGATCTGGGCATTGTGAGCGATGCGAAAGCGGCGCTGACCCTGCTGGTGGAAGTGGCGCAAGAAATGCAGAAAGCGGGCCGCCTGCCGTGCCGCAAAGAATGGGTGGCGGATTGTCAGCAGCGCAAACGCACCCTGCTGCGCAAAACCCATTTTGATAACGTGCCGGTGAAACCGCAGCGCGTGTATGAAGAAATGAACAAAGCGTTTGGCCGCGATGTGTGCTATGTGACCACCATTGGCCTGAGTCAGATTGCGGCCGCGCAGATGCTGCATGTGTTTAAAGATCGCCATTGGATTAATTGTGGCCAAGCGGGCCCGTTAGGTTGGACGATTCCGGCGGCGCTGGGTGTTTGCGCGGCGGATCCGAAACGCAACGTGGTGGCGATTAGCGGCGATTTTGATTTTCAGTTTCTGATTGAAGAACTGGCGGTGGGCGCGCAGTTTAACATTCCGTATATTCATGTGCTGGTGAACAACGCGTATCTGGGCATGATTCGTCAGAGTCAGATGGCGTTTGATCTGGATTATTGCGTGCAGCTGGCGTTTGAAAACATTAACAGCAGCGAAGTGAACGGCTATGGCGTGGATCATGTGAAAGTGGCGGAAGGCCTGGGCTGCAAAGCGATTCGCGTGTTTAAACCGGAAGATATTGCGCCGGCGTTTGAACAAGCGAAAGCGCTGATGGCGCAGTATCGCGTGCCGGTGGTTGTGGAAGTGATTCTGGAACGCGTGACCAACATTAGCATGGGCAGCGAACTGGATAACGTGATGGAATTTGAAGATATCGCCGATAATGCGGCGGATGCGCCGACCGAAACCTGCTTTATGCATTATGAATAA |
| ACPS | ATGACGAGTCCTGTTATTGGCACCCCTTGGAAGAAGCTGAACGCTCCGGTTTCCGAGGAAGCTATCGAAGGCGTGGATAAGTACTGGCGCGCAGCCAACTACCTCTCCATCGGCCAGATCTATCTGCGTAGCAACCCGCTGATGAAGGAGCCTTTCACCCGCGAAGACGTCAAGCACCGTCTGGTCGGTCACTGGGGCACCACCCCGGGCCTGAACTTCCTCATCGGCCACATCAACCGTCTCATTGCTGATCACCAGCAGAACACTGTGATCATCATGGGCCCGGGCCACGGCGGCCCGGCTGGTACCGCTCAGTCCTACCTGGACGGCACCTACACCGAGTACTTCCCGAACATCACCAAGGATGAGGCTGGCCTGCAGAAGTTCTT  CCGCCAGTTCTCCTACCCGGGTGGCATCCCGTCCCACTACGCTCCGGAGACCCCGGGCTCCATCCACGAAGGCGGCGAGCTGGGTTACGCCCTGTCCCACGCCTACGGCGCTGTGATGAACAACCCGAGCCTGTTCGTCCCGGCCATCGTCGGCGACGGCGAAGCTGAGACCGGCCCGCTGGCCACCGGCTGGCAGTCCAACAAGCTCATCAACCCGCGCACCGACGGTATCGTGCTGCCGATCCTGCACCTCAATGGCTACAAGATCGCCAACCCGACCATCCTGTCCCGCATCTCCGACGAAGAGCTCCACGAGTTCTTCCACGGCATGGGCTATGAGCCGTACGAGTTCGTCGCTGGCTTCGACAACGAGGATCACCTGTCGATCCACCGTCGTTTCGCCGAGCTGTTCGAGACCGTCTTCGACGAGATCTGCGACATCAAGGCCGCCGCTCAGACCGACGACATGACTCGTCCGTTCTACCCGATGATCATCTTCCGTACCCCGAAGGGCTGGACCTGCCCGAAGTTCATCGACGGCAAGAAGACCGAGGGCTCCTGGCGTTCCCACCAGGTGCCGCTGGCTTCCGCCCGCGATACCGAGGCCCACTTCGAGGTCCTCAAGAACTGGCTCGAGTCCTACAAGCCGGAAGAGCTGTTCGACGAGAACGGCGCCGTGAAGCCGGAAGTCACCGCCTTCATGCCGACCGGCGAACTGCGCATCGGTGAGAACCCGAACGCCAACGGTGGCCGCATCCGCGAAGAGCTGAAGCTGCCGAAGCTGGAAGACTACGAGGTCAAGGAAGTCGCCGAGTACGGCCACGGCTGGGGCCAGCTCGAGGCCACCCGTCGTCTGGGCGTCTACACCCGCGACATCATCAAGAACAACCCGGACTCCTTCCGTATCTTCGGACCGGATGAGACCGCTTCCAACCGTCTGCAGGCCGCTTACGACGTCACCAACAAGCAGTGGGACGCCGGCTACCTGTCCGCTCAGGTCGACGAGCACATGGCTGTCACCGGCCAGGTCACCGAGCAGCTTTCCGAGCACCAGATGGAAGGCTTCCTCGAGGGCTACCTGCTGACCGGCCGTCACGGCATCTGGAGCTCCTATGAGTCCTTCGTGCACGTGATCGACTCCATGCTGAACCAGCACGCCAAGTGGCTCGAGGCTACCGTCCGCGAGATTCCGTGGCGCAAGCCGATCTCCTCCATGAACCTGCTCGTCTCCTCCCACGTGTGGCGTCAGGATCACAACGGCTTCTCCCACCAGGATCCGGGTGTCACCTCCGTCCTGCTGAACAAGTGCTTCAACAACGATCACGTGATCGGCATCTACTTCCCGGTGGATTCCAACATGCTGCTCGCTGTGGCTGAGAAGTGCTACAAGTCCACCAACAAGATCAACGCCATCATCGCCGGCAAGCAGCCGGCCGCCACCTGGCTGACCCTGGACGAAGCTCGCGCCGAGCTCGAGAAGGGTGCTGCCGAGTGGAAGTGGGCTTCCAACGTGAAGTCCAACGATGAGGCTCAGATCGTGCTCGCCGCCACCGGTGATGTTCCGACTCAGGAAATCATGGCCGCTGCCGACAAGCTGGACGCCATGGGCATCAAGTTCAAGGTCGTCAACGTGGTTGACCTGGTCAAGCTGCAGTCCGCCAAGGAGAACAACGAGGCCCTCTCCGATGAGGAGTTCGCTGAGCTGTTCACCGAGGACAAGCCGGTCCTGTTCGCTTACCACTCCTATGCCCGCGACGTGCGTGGTCTGATCTACGATCGCCCGAACCACGACAACTTCAACGTTCACGGCTACGAGGAGCAGGGCTCCACCACCACCCCGTACGACATGGTTCGCGTGAACAACATCGATCGCTACGAGCTCCAGGCTGAAGCTCTGCGCATGATCGACGCTGACAAGTACGCCGACAAGATCAACGAGCTCGAGGCCTTCCGTCAGGAAGCCTTCCAGTTCGCTGTCGACAACGGCTACGATCACCCGGATTACACCGACTGGGTCTACTCCGGTGTCAACACCAACAAGCAGGGTGCTATCTCCGCTACCGCCGCAACCGCTGGCGATAACGAGTGA |
| PTA | ATGTCCCGTATTATTATGCTGATCCCTACCGGAACCAGCGTCGGTCTGACCAGCGTCAGCCTTGGCGTGATCCGTGCAATGGAACGCAAAGGCGTTCGTCTGAGCGTTTTCAAACCTATCGCTCAGCCGCGTACCGGTGGCGATGCGCCCGATCAGACTACGACTATCGTGCGTGCGAACTCTTCCACCACGACGGCCGCTGAACCGCTGAAAATGAGCTACGTTGAAGGTCTGCTTTCCAGCAATCAGAAAGATGTGCTGATGGAAGAGATCGTCGCAAACTACCACGCTAACACCAAAGACGCTGAAGTCGTTCTGGTTGAAGGTCTGGTCCCGACACGTAAGCACCAGTTTGCCCAGTCTCTGAACTACGAAATCGCTAAAACGCTGAATGCGGAAATCGTCTTCGTTATGTCTCAGGGCACTGACACCCCGGAACAGCTGAAAGAGCGTATCGAACTGACCCGCAACAGCTTCGGCGGTGCCAAAAACACCAACATCACCGGCGTTATCGTTAACAAACTGAACGCACCGGTTGATGAACAGGGTCGTACTCGCCCGGATCTGTCCGAGATTTTCGACGACTCTTCCAAAGCTAAAGTAAACAATGTTGATCCGGCGAAGCTGCAAGAATCCAGCCCGCTGCCGGTTCTCGGCGCTGTGCCGTGGAGCTTTGACCTGATCGCGACTCGTGCGATCGATATGGCTCGCCACCTGAATGCGACCATCATCAACGAAGGCGACATCAATACTCGCCGCGTTAAATCCGTCACTTTCTGCGCACGCAGCATTCCGCACATGCTGGAGCACTTCCGTGCCGGTTCTCTGCTGGTGACTTCCGCAGACCGTCCTGACGTGCTGGTGGCCGCTTGCCTGGCAGCCATGAACGGCGTAGAAATCGGTGCCCTGCTGCTGACTGGCGGTTACGAAATGGACGCGCGCATTTCTAAACTGTGCGAACGTGCTTTCGCTACCGGCCTGCCGGTATTTATGGTGAACACCAACACCTGGCAGACCTCTCTGAGCCTGCAGAGCTTCAACCTGGAAGTTCCGGTTGACGATCACGAACGTATCGAGAAAGTTCAGGAATACGTTGCTAACTACATCAACGCTGACTGGATCGAATCTCTGACTGCCACTTCTGAGCGCAGCCGTCGTCTGTCTCCGCCTGCGTTCCGTTATCAGCTGACTGAACTTGCGCGCAAAGCGGGCAAACGTATCGTACTGCCGGAAGGTGACGAACCGCGTACCGTTAAAGCAGCCGCTATCTGTGCTGAACGTGGTATCGCAACTTGCGTACTGCTGGGTAATCCGGCAGAGATCAACCGTGTTGCAGCGTCTCAGGGTGTAGAACTGGGTGCAGGGATTGAAATCGTTGATCCAGAAGTGGTTCGCGAAAGCTATGTTGGTCGTCTGGTCGAACTGCGTAAGAACAAAGGCATGACCGAAACCGTTGCCCGCGAACAGCTGGAAGACAACGTGGTGCTCGGTACGCTGATGCTGGAACAGGATGAAGTTGATGGTCTGGTTTCCGGTGCTGTTCACACTACCGCAAACACCATCCGTCCGCCGCTGCAGCTGATCAAAACTGCACCGGGCAGCTCCCTGGTATCTTCCGTGTTCTTCATGCTGCTGCCGGAACAGGTTTACGTTTACGGTGACTGTGCGATCAACCCGGATCCGACCGCTGAACAGCTGGCAGAAATCGCGATTCAGTCCGCTGATTCCGCTGCGGCCTTCGGTATCGAACCGCGCGTTGCTATGCTCTCCTACTCCACCGGTACTTCTGGTGCAGGTAGCGACGTAGAAAAAGTTCGCGAAGCAACTCGTCTGGCGCAGGAAAAACGTCCTGACCTGATGATCGACGGTCCGCTGCAGTACGACGCTGCGGTAATGGCTGACGTTGCGAAATCCAAAGCGCCGAACTCTCCGGTTGCAGGTCGCGCTACCGTGTTCATCTTCCCGGATCTGAACACCGGTAACACCACCTACAAAGCGGTACAGCGTTCTGCCGACCTGATCTCCATCGGGCCGATGCTGCAGGGTATGCGCAAGCCGGTTAACGACCTGTCCCGTGGCGCACTGGTTGACGATATCGTCTACACCATCGCGCTGACTGCGATTCAGTCTGCACAGCAGCAGTAA |
| PhaA | ATGAATCGCAGAGTGGCAATCGTCGGCTTCGGCCAGACGGAGATGATGGCGCGCAGCCCGCTGAGCAAGGCGGAGCTAGCCAACCAGGCCGTGCGGCGGGCACTGGAAGATGCGCAGATCACGATGAAGCAGGTCGACGAGATCGTGCTGGCCGACATCGACTACGTGTCGGGCACGGCGGAGTCGGAAATGGAGCTGGCCGACTGGGTCGGGCACCGCCGCAAGCCCGTGGTCAAGATCGAAACCGGCGGCACCGTGGGCGGGTCGGCGGCGCTGTCGGCTGTACACCATATCGCAGCCGGCGCCTGTGACGTCGCGCTGGTGTCGGCAACGGCGAAGTTCGTCGGCCCGCCGCCCGGCACGCTGCCGCGCGGGCCGTTCCTGCAGGCGGCGATCAATTCCGGCCTGCACGCGCTCACCGAAAAATGGTGCAGCGTGGGCGCCGTGGGGACCTTTTCCCTGATGGCGAGCTCCTATGTCAAGCTGTCCGGCTGCACCGAGGAAGCGGTTGCCATCGCCCGGGTCAAGGCCGCCAACAACGCGCTTAAAAACCCCTATGCGCATCTGCGCGAGCACCTGACCGTGGAAGACGTGCTCAACTCGCCGATGCTGACGGCGCCGATGCGCCAGCTCCATATGTGCCCGATCACCGAAGGCTCGGCGGCGATGATCTTCGCCAGCGAGGACGTGGCGCACAAGCTCACCAAAAAGCCGGTATGGGTGAAGGACATGGTCACCATCCACTCCGCGCAGCACTGGGCCGCGCTGCAGGACTTCATCGCCCCCAGGGAGCGTTGCGTGCTGCCGAGCCTGGCCAAGGCGTGCGAAGTGCTCTACAAGCGCAACGGCATCACGCATCCCGCCAAACAGCTCGATGTGATCGAGATGTACGAGCCCTGCACTTGGGCCGAACTGGTGTGGATGGAAACCATGGGCCTGTGCGAGCCGAACCAGGCGTGGAAGCTGATGGGCACCGGCGCCACCGATATCGATGGCGAACTGCCAATCAATCCATCAGGCGGGGTCACGTGTACCAACCCGGGCGTTCCCTCGACCCTGCTGCGTTATGGCGAACTGGCCCTGCAGATCCGCGGCGATGCCGGCGAGCACCAGGTCCCGCGCGATGTCAGGCTTGGCCTGGCCACCGGCTTCGGCGGCACCGGCTGGACGCCGCTGATGCTGCTGTCCAAGGACAAGTAA |
| Hmgs | ATGACAATTGGGATTGATAAAATTAGTTTTTTTGTGCCCCCTTATTATATTGATATGACGGCACTGGCTGAAGCCAGAAATGTAGACCCTGGAAAATTTCATATTGGTATTGGGCAAGACCAAATGGCGGTGAACCCAATCAGCCAAGATATTGTGACATTTGCAGCCAATGCCGCAGAAGCGATCTTGACCAAAGAAGATAAAGAGGCCATTGATATGGTGATTGTCGGGACTGAGTCCAGTATCGATGAGTCAAAAGCGGCCGCAGTTGTCTTACATCGTTTAATGGGGATTCAACCTTTCGCTCGCTCTTTCGAAATCAAGGAAGGTTGTTACGGAGCAACAGCAGGCTTACAGTTAGCTAAGAATCACGTAGCCTTACATCCAGATAAAAAAGTCTTGGTCGTAGCGGCAGATATTGCAAAATATGGCTTAAATTCTGGCGGTGAGCCTACACAAGGAGCTGGGGCGGTTGCAATGTTAGTTGCTAGTGAACCGCGCATTTTGGCTTTAAAAGAGGATAATGTGATGCTGACGCAAGATATCTATGACTTTTGGCGTCCAACAGGCCACCCGTATCCTATGGTCGATGGTCCTTTGTCAAACGAAACCTACATCCAATCTTTTGCCCAAGTCTGGGATGAACATAAAAAACGAACCGGTCTTGATTTTGCAGATTATGATGCTTTAGCGTTCCATATTCCTTACACAAAAATGGGCAAAAAAGCCTTATTAGCAAAAATCTCCGACCAAACTGAAGCAGAACAGGAACGAATTTTAGCCCGTTATGAAGAAAGCATCATCTATAGTCGTCGCGTAGGAAACTTGTATACGGGTTCACTTTATCTGGGACTCATTTCCCTTTTAGAAAATGCAACGACTTTAACCGCAGGCAATCAAATTGGGTTATTCAGTTATGGTTCTGGTGCTGTCGCTGAATTTTTCACTGGTGAATTAGTAGCTGGTTATCAAAATCATTTACAAAAAGAAACTCATTTAGCACTGCTGGATAATCGGACAGAACTTTCTATCGCTGAATATGAAGCCATGTTTGCAGAAACTTTAGACACAGACATTGATCAAACGTTAGAAGATGAATTAAAATATAGTATTTCTGCTATTAATAATACCGTTCGTTCTTATCGAAACTAA |
| Hmgr | ATGAAAACAGTAGTTATTATTGATGCATTACGAACACCAATTGGAAAATATAAAGGCAGCTTAAGTCAAGTAAGTGCCGTAGACTTAGGAACACATGTTACAACACAACTTTTAAAAAGACATTCCACTATTTCTGAAGAAATTGATCAAGTAATCTTTGGAAATGTTTTACAAGCTGGAAATGGCCAAAATCCCGCACGACAAATAGCAATAAACAGCGGTTTGTCTCATGAAATTCCCGCAATGACGGTTAATGAGGTCTGCGGATCAGGAATGAAGGCCGTTATTTTGGCGAAACAATTGATTCAATTAGGAGAAGCGGAAGTTTTAATTGCTGGCGGGATTGAGAATATGTCCCAAGCACCTAAATTACAACGATTTAATTACGAAACAGAAAGCTACGATGCGCCTTTTTCTAGTATGATGTACGATGGGTTAACGGATGCCTTTAGTGGTCAAGCAATGGGCTTAACTGCTGAAAATGTGGCCGAAAAGTATCATGTAACTAGAGAAGAGCAAGATCAATTTTCTGTACATTCACAATTAAAAGCAGCTCAAGCACAAGCAGAAGGGATATTCGCTGACGAAATAGCCCCATTAGAAGTGTCAGGAACGCTTGTGGAGAAAGATGAAGGGATTCGCCCTAATTCGAGCGTTGAGAAGCTAGGAACGCTTAAAACAGTTTTTAAAGAAGACGGTACTGTAACAGCAGGGAATGCATCAACCATTAATGATGGGGCTTCTGCTTTGATTATTGCTTCACAAGAATATGCCGAAGCACACGGTCTTCCTTATTTAGCTATTATTCGAGACAGTGTGGAAGTCGGTATTGATCCAGCCTATATGGGAATTTCGCCGATTAAAGCCATTCAAAAACTGTTAGCGCGCAATCAACTTACTACGGAAGAAATTGATCTGTATGAAATCAACGAAGCATTTGCAGCAACTTCAATCGTGGTCCAAAGAGAACTGGCTTTACCAGAGGAAAAGGTCAACATTTATGGTGGCGGTATTTCATTAGGTCATGCGATTGGTGCCACAGGTGCTCGTTTATTAACGAGTTTAAGTTATCAATTAAATCAAAAAGAAAAGAAATATGGAGTGGCTTCTTTATGTATTGGCGGTGGCTTAGGACTCGCTATGCTACTAGAGAGACCTCAGCAAAAAAAAAACAGCCGATTTTATCAAATGAGTCCTGAGGAACGCCTGGCTTCTCTTCTTAATGAAGGCCAGATTTCTGCTGATACAAAAAAAGAATTTGAAAATACGGCTTTATCTTCGCAGATTGCCAATCATATGATTGAAAATCAAATCAGTGAAACAGAAGTGCCGATGGGCGTTGGCTTACATTTAACAGTGGACGAAACTGATTATTTGGTACCAATGGCGACAGAAGAGCCCTCAGTGATTGCGGCTTTGAGTAATGGTGCAAAAATAGCACAAGGATTTAAAACAGTGAATCAACAACGCTTAATGCGTGGACAAATCGTTTTTTACGATGTTGCAGATCCCGAGTCATTGATTGATAAACTACAAGTAAGAGAAGCGGAAATTTTTCAACAAGCAGAGTTAAGTTATCCATCTATCGTAAAACGGGGCGGCGGCTTAAGAGATTTGCAGTATCGTGCTTTTGATGAATCATTTATATCTGTCGACTTTTTAGTAGATGTTAAGGATGCAATGGGGGCAAATATCGTTAACGCTATGTTGGAAGGTGTGGTCGAGTTGTTCCGTGAATGGTTTGCGGAGCAAAAAATTTTATTCAGTATTTTAAGTAATTATGCCACGGAGTCGGTTGTTACGATGAAAACGGCTATTCCAGTTTCACGTTTAAGTAAGGGGAGCAATGGCCGGGAAATTGCTGAAAAAATTGTTTTAGCTTCACGCTATGCTTCATTAGATCCTTATCGGGCAGTCACGCATAACAAAGGAATCATGAATGGCATTGAAGCTGTAGTTTTAGCTACAGGAAATGATACACGCGCTGTTAGCGCTTCTTGTCATGCTTTTGCGGTGAAGGAAGGTCGCTACCAAGGCTTGACTAGTTGGACGCTGGATGGCGAACAACTAATTGGTGAAATTTCAGTTCCGCTTGCTTTAGCCACGGTTGGCGGTGCCACAAAAGTCTTACCTAAATCTCAAGCAGCCGCTGATTTGTTAGCAGTGACGGATGCAAAAGAACTAAGTCGAGTAGTAGCGGCTGTTGGTTTGGCACAAAATTTAGCGGCGTTACGGGCCTTAGTCTCTGAAGGAATTCAAAAAGGACACATGGCTCTACAAGCACGTTCTTTAGCGATGACGGTCGGAGCTACTGGTAAAGAAGTTGAGGCAGTCGCTCAACAATTAAAACGTCAAAAAACGATGAACCAAGACCGAGCCTTGGCTATTTTAAATGATTTAAGAAAACAATAA |
| Mvk | ATGGTTTCATGTTCTGCGCCCGGGAAAATCTATCTTTTCGGAGAACATGCGGTTGTTTACGGAGAAACCGCAATAGCGTGTGCAGTAGAGTTAAGGACCCGGGTGCGGGCGGAGTTAAATGACTCCATAACTATCCAGTCTCAGATCGGCAGGACAGGTCTTGATTTTGAAAAACATCCCTATGTCTCTGCAGTGATTGAAAAAATGAGAAAATCTATCCCCATAAATGGTGTTTTTTTAACTGTTGATTCCGACATTCCTGTTGGGTCAGGGCTCGGCTCATCTGCTGCTGTTACGATTGCAAGCATAGGAGCTCTCAACGAACTTTTCGGATTCGGGCTTTCGCTTCAGGAAATTGCGAAACTGGGGCATGAAATTGAGATAAAAGTTCAGGGTGCAGCGAGCCCTACTGACACCTATGTTTCTACTTTCGGAGGAGTCGTTACCATCCCTGAAAGGAGAAAGCTTAAGACTCCTGACTGTGGAATTGTTATAGGGGACACCGGAGTTTTTTCTTCTACAAAAGAGCTTGTGGCAAACGTCAGGCAGCTCCGCGAAAGTTACCCTGATCTTATCGAACCTCTTATGACTTCTATTGGCAAAATCTCCAGAATCGGTGAGCAACTTGTACTTTCCGGGGACTATGCTTCTATTGGCAGGCTTATGAATGTAAATCAGGGACTGCTTGATGCACTTGGAGTTAATATCCTTGAGCTTTCACAGCTTATCTATTCTGCAAGGGCAGCAGGAGCTTTCGGGGCAAAAATTACTGGAGCGGGAGGCGGTGGTTGTATGGTTGCGCTAACTGCACCGGAGAAATGTAATCAGGTAGCGGAAGCCATTGCAGGTGCGGGGGGCAAAGTGACCATTACAAAACCTACGGAACAGGGGTTGAAGGTCGATTGA |
| Pmvk | ATGATTGCTGTTAAAACTTGCGGAAAACTCTATTGGGCAGGTGAATATGCTATTTTAGAGCCAGGGCAGTTAGCTTTGATAAAGGATATTCCCATCTATATGAGGGCTGAGATTGTTTTTTCTGACAGCTACCGTATCTATTCAGATATGTTTGATTTCGCAGTGGACTTAAGGCCTAATCCTGACTACAGCTTGATTCAAGAAACGATTGCTTTGATGGGAGACTTCCTCGCTGTTCGTGGTCAGAATTTAAGACCTTTTTCTCTAGAAATCTGTGGCAAAATGGAACGAGAAGGGAAAAAGTTTGGTCTAGGTTCTAGTGGCAGCGTCGTTGTCTTGGTTGTCAAGGCTTTACTGGCTCTGTATGATGTTTCTGTTGATCAGGAGCTCTTGTTCAAGCTGACTAGCGCTGTCTTGCTCAAGCGAGGAGACAATGGTTCCATGGGAGACCTTGCCTGTATTGTGGCAGAGGATTTGGTTCTCTACCAGTCATTTGATCGCCAGAAGGTGGCTGCTTGGTTAGAAGAAGAAAACTTGGCGACAGTTCTGGAGCGTGATTGGGGCTTTTCAATTTCACAAGTGAAACCAACTTTAGAATGTGATTTCTTAGTGGGATGGACCAAGGAAGTGGCTGTATCGAGTCACATGGTCCAGCAAATCAAGCAAAATATCAATCAAAATTTTTTAAGTTCCTCAAAAGAAACGGTGGTTTCTTTGGTCGAAGCCTTGGAGCAGGGGAAAGCCGAAAAAGTTATCGAGCAAGTAGAAGTAGCCAGCAAGCTTTTAGAAGGCTTGAGTACAGATATTTACACGCCTTTGCTTAGACAGTTGAAAGAAGCCAGTCAAGATTTGCAGGCCGTTGCCAAGAGTAGTGGTGCTGGTGGTGGTGACTGTGGCATCGCCTTGAGTTTTGATGCGCAATCAACCGAAACCTTAAAAAATCGTTGGGCCGATCTGGGGATTGAGCTCTTATATCAAGAAAGGATAGGACATGACGACAAATCGTAA |
| Mdc | ATGGATAGAGAGCCTGTAACAGTACGTTCCTACGCAAATATTGCTATTATCAAATATTGGGGAAAGAAAAAAGAAAAAGAGATGGTGCCTGCTACTAGCAGTATTTCTCTAACTTTGGAAAATATGTATACAGAGACGACCTTGTCGCCTTTACCAGCCAATGTAACAGCTGACGAATTTTACATCAATGGTCAGCTACAAAATGAGGTCGAGCATGCTAAGATGAGTAAGATTATTGACCGTTATCGTCCAGCTGGTGAGGGCTTTGTCCGTATCGATACTCAAAACAATATGCCTACTGCAGCGGGCCTGTCCTCAAGTTCTAGTGGTTTGTCCGCCCTGGTCAAGGCTTGTAATGCTTATTTCAAGCTTGGATTGGATAGAAGTCAGTTGGCACAGGAAGCCAAATTTGCCTCAGGCTCTTCTTCTCGGAGTTTTTATGGACCACTAGGAGCCTGGGATAAGGATAGTGGAGAAATTTACCCTGTAGAGACAGACTTGAAACTAGCTATGATTATGTTGGTGCTAGAGGACAAGAAAAAACCAATCTCTAGCCGTGACGGGATGAAACTTTGTGTGGAAACCTCGACGACTTTTGACGACTGGGTTCGTCAGTCTGAGAAGGACTATCAGGATATGCTGATTTATCTCAAGGAAAATGATTTTGCCAAGATTGGAGAATTAACGGAGAAAAATGCCCTGGCTATGCATGCTACGACAAAGACTGCTAGTCCAGCCTTTTCTTATCTGACGGATGCCTCTTATGAGGCTATGGACTTTGTCCGCCAGCTTCGTGAGAAAGGAGAGGCCTGCTACTTTACCATGGATGCTGGTCCCAATGTTAAGGTCTTCTGTCAGGAGAAAGACTTGGAGCATTTATCAGAAATTTTCGGTCAGCGTTATCGCTTGATTGTGTCAAAAACAAAGGATTTGAGTCAAGATGATTGCTGTTAA |
| IDI | ATGCAGACCGAACATGTGATTCTGCTGAACGCGCAAGGCGTGCCGACCGGCACCCTGGAAAAATATGCGGCGCATACCGCGGATACCCGCCTGCATCTGGCGTTTAGCAGCTGGCTGTTTAACGCGAAAGGTCAGCTGCTGGTGACCCGCCGCGCGCTGAGCAAAAAAGCGTGGCCGGGCGTGTGGACCAACAGCGTGTGCGGCCATCCGCAGCTGGGCGAAAGCAACGAAGATGCGGTGATTCGCCGCTGCCGCTATGAACTGGGCGTGGAAATTACCCCGCCGGAAAGCATTTATCCGGATTTTCGCTATCGCGCGACCGATCCGAGCGGCATTGTGGAAAACGAAGTGTGCCCGGTGTTTGCGGCGCGCACCACGAGCGCGCTGCAGATTAACGATGATGAAGTGATGGATTATCAGTGGTGCGATCTGGCGGATGTGCTGCATGGCATTGATGCGACCCCGTGGGCGTTTAGCCCGTGGATGGTGATGCAAGCGACCAACCGCGAAGCGCGCAAACGCCTGAGCGCGTTTACGCAGCTGAAATAA |
| ISPA | ATGGATTTTCCGCAGCAGCTGGAAGCGTGCGTGAAACAAGCGAACCAAGCGCTGAGCCGCTTTATTGCGCCGCTGCCGTTTCAGAACACCCCGGTGGTGGAAACCATGCAGTATGGCGCGCTGCTGGGCGGCAAACGCCTGCGCCCGTTTCTGGTGTATGCGACCGGCCACATGTTTGGCGTGAGCACCAACACCCTGGATGCGCCGGCGGCCGCGGTGGAATGCATTCATGCGTATAGCCTGATTCATGATGATTTACCGGCGATGGATGACGATGATCTGCGTCGCGGCCTGCCGACCTGCCATGTGAAATTTGGCGAAGCGAACGCGATTCTGGCGGGCGATGCGCTGCAGACCCTGGCGTTTAGCATTCTGAGCGATGCGGATATGCCGGAAGTGAGCGATCGCGATCGCATTAGCATGATTAGCGAACTGGCGAGCGCGAGCGGCATTGCGGGCATGTGCGGCGGCCAAGCGCTGGATCTGGATGCGGAAGGCAAACATGTGCCGCTGGATGCGCTGGAACGCATTCACCGCCACAAAACCGGCGCCTTAATTCGTGCCGCCGTGCGCTTAGGCGCGCTGAGCGCGGGTGATAAAGGCCGCCGCGCGCTGCCGGTGCTGGATAAATATGCGGAAAGCATTGGCCTGGCGTTTCAAGTGCAAGATGATATTCTGGATGTGGTGGGCGATACCGCGACCCTGGGCAAACGCCAAGGCGCGGATCAGCAGCTGGGCAAAAGCACCTATCCGGCGCTGCTGGGTTTAGAACAAGCGCGCAAAAAAGCGCGCGATCTGATTGATGATGCGCGTCAGAGCCTGAAACAGCTGGCGGAACAGAGCCTGGATACGAGCGCGCTGGAAGCGCTGGCGGATTATATTATTCAGCGCAACAAATAA |
| MdFs | ATGGAATTTCGCGTGCATCTGCAAGCGGATAACGAACAGAAAATTTTTCAGAATCAGATGAAACCGGAACCGGAAGCGAGCTATCTGATTAATCAGCGCCGCAGCGCGAACTATAAACCGAACATTTGGAAAAACGATTTTCTGGATCAGAGCCTGATTAGCAAATATGATGGCGATGAATATCGCAAACTGAGCGAAAAACTGATTGAAGAAGTGAAAATTTATATTAGCGCGGAAACCATGGATCTGGTGGCGAAACTGGAACTGATTGATAGCGTGCGCAAACTGGGCCTGGCGAACCTGTTTGAAAAAGAAATTAAAGAAGCGCTGGATAGCATTGCGGCGATTGAAAGCGATAACCTGGGCACCCGCGATGATCTGTATGGCACCGCGCTGCATTTTAAAATTCTGCGTCAGCATGGCTATAAAGTGAGCCAAGATATTTTTGGCCGCTTTATGGATGAAAAAGGCACCCTGGAAAACCATCATTTTGCGCATCTGAAAGGCATGCTGGAACTGTTTGAAGCGAGCAACCTGGGCTTTGAAGGCGAAGATATTCTGGATGAAGCGAAAGCGAGCCTGACCCTGGCGCTGCGCGATAGCGGCCATATTTGCTATCCGGATAGCAACCTGAGCCGCGATGTGGTGCATAGCCTGGAACTGCCGAGCCATCGCCGCGTGCAGTGGTTTGATGTGAAATGGCAGATTAACGCGTATGAAAAAGATATTTGCCGCGTGAACGCGACCCTGCTGGAACTGGCGAAACTGAACTTTAACGTGGTGCAAGCGCAGCTGCAGAAAAACCTGCGCGAAGCGAGCCGCTGGTGGGCGAACCTGGGCATTGCGGATAACCTGAAATTTGCGCGCGATCGCCTGGTGGAATGCTTTGCGTGCGCGGTGGGCGTGGCGTTTGAACCGGAACATAGCAGCTTTCGCATTTGCCTGACCAAGGTGATTAACCTGGTGCTGATTATTGATGATGTGTATGATATTTATGGCAGCGAAGAGGAACTGAAACATTTTACCAACGCGGTGGATCGCTGGGATAGCCGCGAAACCGAACAGCTGCCGGAATGCATGAAAATGTGCTTTCAAGTGCTGTATAACACCACCTGCGAAATTGCGCGCGAAATTGAAGAGGAAAACGGCTGGAACCAAGTGCTGCCGCAGCTGACCAAAGTGTGGGCCGATTTTTGCAAAGCGCTGCTGGTGGAAGCGGAATGGTATAACAAAAGCCATATTCCGACCCTGGAAGAATATCTGCGCAACGGCTGCATTAGCAGTAGCGTGAGCGTGCTGCTGGTGCATAGCTTTTTTAGCATTACCCATGAAGGCACCAAAGAAATGGCGGATTTTCTGCATAAAAACGAAGATCTGTTATATAACATTAGCCTGATTGTGCGCCTGAACAACGATCTGGGCACGAGCGCGGCGGAACAAGAACGCGGCGATAGCCCGAGCAGCATTGTGTGCTATATGCGCGAAGTGAACGCGAGCGAAGAAACCGCGCGCAAAAACATTAAAGGCATGATTGATAACGCGTGGAAAAAAGTGAACGGCAAATGCTTTACCACCAACCAAGTGCCGTTTCTGAGCAGCTTTATGAACAACGCGACCAACATGGCGCGCGTGGCGCATAGCCTGTATAAAGATGGCGATGGCTTTGGCGATCAAGAAAAAGGCCCGCGCACCCATATTCTGAGCCTGCTGTTTCAGCCGCTGGTGAACTAA |
| SUMO-AFS | ATGAGCGGCATGAGCGATAGCGAAGTGAACCAAGAAGCGAAGCCGGAAGTGAAACCGGAAGTGAAGCCGGAAACCCATATTAACCTGAAAGTGAGCGATGGCAGCAGCGAAATTTTTTTTAAAATTAAAAAAACCACCCCGCTGCGCCGCCTGATGGAAGCGTTTGCGAAACGCCAAGGCAAAGAAATGGATAGCCTGCGCTTTCTGTATGATGGCATTCGCATTCAAGCGGATCAGACCCCGGAAGATCTGGATATGGAAGATAACGATATTATTGAAGCGCATCGCGAACAGATTGGCGGCGAAATTGATGCGATGCTGCATCAGACCGGCGGCATGGAATTTCGCGTGCATCTGCAAGCGGATAACGAACAGAAAATTTTTCAGAATCAGATGAAACCGGAACCGGAAGCGAGCTATCTGATTAATCAGCGCCGCAGCGCGAACTATAAACCGAACATTTGGAAAAACGATTTTCTGGATCAGAGCCTGATTAGCAAATATGATGGCGATGAATATCGCAAACTGAGCGAAAAACTGATTGAAGAAGTGAAAATTTATATTAGCGCGGAAACCATGGATCTGGTGGCGAAACTGGAACTGATTGATAGCGTGCGCAAACTGGGCCTGGCGAACCTGTTTGAAAAAGAAATTAAAGAAGCGCTGGATAGCATTGCGGCGATTGAAAGCGATAACCTGGGCACCCGCGATGATCTGTATGGCACCGCGCTGCATTTTAAAATTCTGCGTCAGCATGGCTATAAAGTGAGCCAAGATATTTTTGGCCGCTTTATGGATGAAAAAGGCACCCTGGAAAACCATCATTTTGCGCATCTGAAAGGCATGCTGGAACTGTTTGAAGCGAGCAACCTGGGCTTTGAAGGCGAAGATATTCTGGATGAAGCGAAAGCGAGCCTGACCCTGGCGCTGCGCGATAGCGGCCATATTTGCTATCCGGATAGCAACCTGAGCCGCGATGTGGTGCATAGCCTGGAACTGCCGAGCCATCGCCGCGTGCAGTGGTTTGATGTGAAATGGCAGATTAACGCGTATGAAAAAGATATTTGCCGCGTGAACGCGACCCTGCTGGAACTGGCGAAACTGAACTTTAACGTGGTGCAAGCGCAGCTGCAGAAAAACCTGCGCGAAGCGAGCCGCTGGTGGGCGAACCTGGGCATTGCGGATAACCTGAAATTTGCGCGCGATCGCCTGGTGGAATGCTTTGCGTGCGCGGTGGGCGTGGCGTTTGAACCGGAACATAGCAGCTTTCGCATTTGCCTGACCAAAGTGATTAACCTGGTGCTGATTATTGATGATGTGTATGATATTTATGGCAGCGAAGAGGAACTGAAACATTTTACCAACGCGGTGGATCGCTGGGATAGCCGCGAAACCGAACAGCTGCCGGAATGCATGAAAATGTGCTTTCAAGTGCTGTACAATACCACCTGCGAAATTGCGCGCGAAATTGAAGAGGAAAACGGCTGGAACCAAGTGCTGCCGCAGCTGACGAAAGTGTGGGCGGATTTTTGCAAAGCGCTGCTGGTGGAAGCGGAATGGTATAACAAAAGCCATATTCCGACCCTGGAAGAATATCTGCGCAACGGCTGCATTAGCAGTAGCGTGAGCGTGCTGCTGGTGCATAGCTTTTTTAGCATTACCCATGAAGGCACCAAAGAAATGGCGGATTTTCTGCATAAAAACGAAGATCTGCTGTATAACATTAGCCTGATTGTGCGCCTGAACAACGATCTGGGCACGAGCGCGGCGGAACAAGAACGCGGCGATAGCCCGAGCAGCATTGTGTGCTATATGCGCGAAGTGAACGCGAGCGAAGAAACCGCGCGCAAAAACATTAAAGGCATGATTGATAACGCGTGGAAAAAAGTGAACGGCAAATGCTTTACCACCAACCAAGTGCCGTTTCTGAGCAGCTTTATGAACAACGCGACCAACATGGCGCGCGTGGCGCATAGCCTGTATAAAGATGGCGATGGCTTTGGCGATCAAGAAAAAGGCCCGCGCACCCATATTCTGAGCCTGCTGTTTCAGCCGCTGGTGAACTAA |

**References**

1. Sundaram S, Diehl C, Cortina NS, Bamberger J, Paczia N. Erb TJ. A modular *in vitro* platform for the production of terpenes and polyketides from CO_2_*.* *Angew Chem Int Ed Engl*. 2021;60(30):16420-16425.

2. Korman TP, Opgenorth PH. Bowie JU. A synthetic biochemistry platform for cell free production of monoterpenes from glucose*.* *Nat Commun*. 2017;8:15526.

3. Durbecq V, Sainz G, Oudjama Y, Clantin B, Bompard-Gilles C, Tricot C, Caillet J, Stalon V, Droogmans L. Villeret V. Crystal structure of isopentenyl diphosphate:dimethylallyl diphosphate isomerase*.* *The EMBO Journal*. 2001;20(7):1530-1537.
